# Rapid and Accurate Screening of the COF Space for Natural Gas Purification: COFInformatics

**DOI:** 10.1021/acsami.4c01641

**Published:** 2024-04-08

**Authors:** Gokhan Onder Aksu, Seda Keskin

**Affiliations:** Department of Chemical and Biological Engineering, Koc University, Rumelifeneri Yolu, Sariyer, 34450 Istanbul, Turkey

**Keywords:** covalent organic frameworks, machine learning, CO_2_/CH_4_ separation, molecular simulation, adsorption

## Abstract

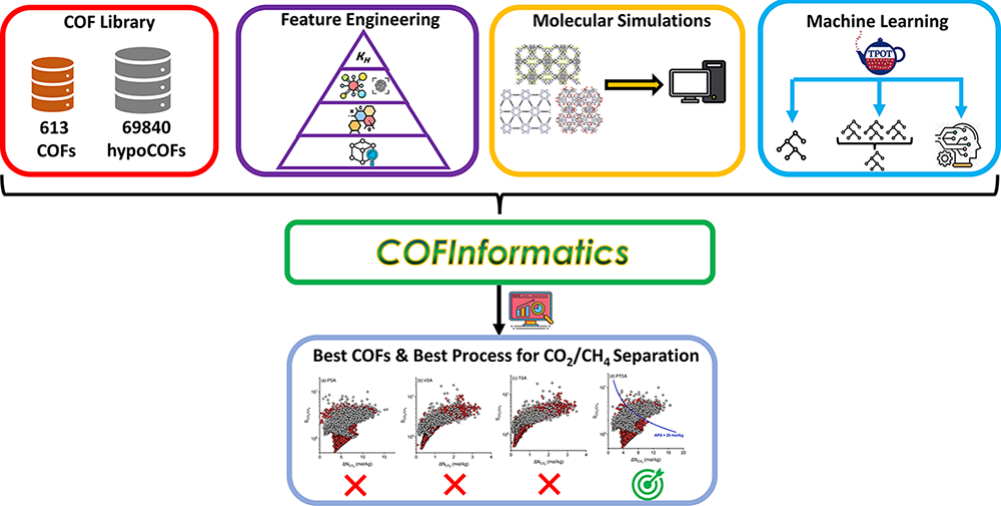

In this work, we introduced COFInformatics, a computational approach merging molecular simulations and machine learning (ML) algorithms, to evaluate all synthesized and hypothetical covalent organic frameworks (COFs) for the CO_2_/CH_4_ mixture separation under four different adsorption-based processes: pressure swing adsorption (PSA), vacuum swing adsorption (VSA), temperature swing adsorption (TSA), and pressure–temperature swing adsorption (PTSA). We first extracted structural, chemical, energy-based, and graph-based molecular fingerprint features of every single COF structure in the very large COF space, consisting of nearly 70,000 materials, and then performed grand canonical Monte Carlo simulations to calculate the CO_2_/CH_4_ mixture adsorption properties of 7540 COFs. These features and simulation results were used to develop ML models that accurately and rapidly predict CO_2_/CH_4_ mixture adsorption and separation properties of all 68,614 COFs. The most efficient separation process and the best adsorbent candidates among the entire COF spectrum were identified and analyzed in detail to reveal the most important molecular features that lead to high-performance adsorbents. Our results showed that (i) many hypoCOFs outperform synthesized COFs by achieving higher CO_2_/CH_4_ selectivities; (ii) the top COF adsorbents consist of narrow pores and linkers comprising aromatic, triazine, and halogen groups; and (iii) PTSA is the most efficient process to use COF adsorbents for natural gas purification. We believe that COFInformatics promises to expedite the evaluation of COF adsorbents for CO_2_/CH_4_ separation, thereby circumventing the extensive, time- and resource-intensive molecular simulations.

## 1 Introduction

Currently, fossil fuels are the source of 80% of the world’s energy.^1^ This reliance on fossil fuels has led to a significant increase in atmospheric CO_2_ levels, a key contributor to the greenhouse effect. To address issues regarding the rapid changes in the Earth’s atmosphere, various CO_2_ capture and storage (CCS) methods have been suggested.^2^ These methods encompass the entire spectrum of fossil fuel use, including precombustion treatment, optimizing the combustion process, and capturing CO_2_ after postcombustion. Among these, separating CO_2_ from natural gas, primarily composed of CH_4_ (75–90%), is crucial for large-scale industrial applications because CO_2_ does not only cause corrosion in the pipelines carrying the natural gas but also reduces its energy content.^3^ Adsorption is an energy-efficient separation method that utilizes porous materials to selectively capture CO_2_ from CH_4_. Since the traditional adsorbents such as zeolites and activated carbons generally suffer from either low selectivities or low regenerabilities,^4^ discovering new adsorbent materials for efficient separation of CO_2_/CH_4_ mixtures is critical.

Covalent organic frameworks (COFs) are mechanically, thermally, and chemically robust materials, emerged as promising adsorbents thanks to their exceptional structural characteristics such as high porosities, large surface areas, and low densities.^5−7^ These properties, along with their diverse chemical compositions, make COFs suitable for a wide range of uses like gas storage, separation, catalysis, and energy storage.^8,9^ Experimentally discovered COFs were collected into two computation-ready databases: Computation-Ready Experimental COF (CoRE COF) database^10−12^ containing 613 COFs, and the Clean, Uniform, and Refined with Automatic Tracking from Experimental Database (CURATED COFs)^13,14^ which originally includes 648 structurally optimized COFs. Alongside the synthesized COFs, Smit’s group constructed the first hypothetical, computer-generated COF (hypoCOF) database, which consists of 69,840 different COF structures.^15,16^ With the generation of other hypothetical databases such as Genomic COF,^17,18^ and ReDD-COFFEE^19^ databases, a very large material spectrum composed of various types of structures has emerged. Examining this large material space using only experimental methods to identify the most promising adsorbents is simply impossible. High-throughput computational screening (HTCS) approaches, which basically rely on molecular simulations, efficiently evaluate the materials’ gas adsorption and separation capabilities.^20^ These computational approaches do not only direct the experimental efforts toward the most useful materials but also pinpoint the hypothetical materials that have the potential to surpass performance of the synthesized ones.^21,22^

Tong et al.^10^ examined 187 CoRE COFs using grand canonical Monte Carlo (GCMC) simulations for the separation of noble gases at pressure swing adsorption (PSA) and vacuum swing adsorption (VSA) processes and demonstrated that COFs can achieve high Kr/Ar, Xe/Kr, and Rn/Xe selectivities. Lan et al.^23^ investigated the same set of CoRE COFs using GCMC simulations to compute the iodine and methyl iodide adsorption capacities and reported that these COFs were more effective than the traditional adsorbents like activated carbon, alumina, and zeolites. Smit’s group screened 296 CURATED COFs for CO_2_/N_2_ separation for a pressure–temperature swing adsorption (PTSA) process using GCMC simulations and identified the most effective COF adsorbents with the highest CO_2_ working capacities and the lowest parasitic energies.^13^ Our group studied both CoRE COF and CURATED COF databases to examine adsorption-based CO_2_/H_2_,^24^ CO_2_/N_2_,^25^ CH_4_/H_2_, CH_4_/N_2_, C_2_H_6_/CH_4_^26^ separations and H_2_S + CO_2_ capture from CH_4_^27^ using GCMC simulations.

In addition to these computational studies on the synthesized COFs, a small number of molecular simulation studies focused on hypothetical COFs: Mercado and co-workers screened a database comprising 69,840 computer-generated materials for CH_4_ storage and showed that 304 hypoCOFs surpass the traditional adsorbents in terms of deliverable CH_4_ capacities.^16^ They also investigated the same database for CO_2_/N_2_ separation to identify nearly 400 hypoCOFs with lower parasitic energy compared to the conventional amine scrubbing process for CO_2_ capture.^15^ Our group further explored this hypoCOF database for CO_2_/H_2_ separation at PSA and VSA conditions and discovered that hypoCOFs can attain significantly higher CO_2_/H_2_ selectivities, outperforming the synthesized COF adsorbents.^28^

While numerous extensive HTCS studies have been conducted on the adsorption-based CO_2_/N_2_, CO_2_/H_2_, and CH_4_/H_2_ separations using COFs, research focusing on CO_2_/CH_4_ separation is relatively scarce in the literature: A total of 46 COFs were evaluated for CO_2_/CH_4_ separation under PSA condition using GCMC simulations, and the top COFs with high CO_2_ working capacities and selectivities were found to be comparable with zeolites.^29^ Yan et al.^12^ screened 298 CoRE COFs for membrane-based CO_2_/CH_4_ separation utilizing MD simulations and uncovered that fluorine and chlorine groups in the COFs enhance membrane selectivities. As this literature review highlights, there is a strong need to explore the very large material space of COFs, both synthesized and hypothetical structures, for CO_2_/CH_4_ separation under various adsorption-based separation process conditions to identify the most efficient adsorbents and process types.

Nevertheless, using HTCS to study the entire COF material spectrum comprising thousands of materials is computationally expensive. Recently, machine learning (ML) combined with HTCS has been employed to rapidly predict gas adsorption and separation capabilities of a vast array of COFs.^30,31^ For example, Pardakhti et al.^32^ applied ML algorithms to estimate CH_4_ storage capabilities of 69,839 hypoCOFs and demonstrated that incorporating chemical and structural features as inputs in an ML model results in much precise CH_4_ uptake predictions. Fanourgakis et al.^33^ employed a self-consistent ML approach to decrease the cost of molecular simulations and identified the top COFs for CH_4_ storage among 69,840 hypoCOFs. Cao et al.^34^ integrated ML algorithms with molecular simulations to screen CoRE COFs and hypoCOFs for adsorption-based C_2_H_6_/C_2_H_4_ separation. The same group also developed ML models to predict i-C_4_H_8_ permeabilities and membrane selectivities of COFs for separation of i-C_4_H_8_/C_4_H_6_ mixture and revealed that pore size and porosity are very critical in determining COF membranes’ separation performance.^35^ Recently, our group developed ML models to screen CoRE COFs and hypoCOFs for CH_4_/H_2_ separation and showed that pore size and heat of adsorption of gases are the main factors determining the separation performance of COFs.^36^

Motivated from the great potential of harnessing HTCS and ML methods to examine a large number and variety of materials, in this work, we evaluated all synthesized and hypothetical COFs, 613 CoRE COFs and 69,840 hypoCOFs, for CO_2_/CH_4_ separation under four different processes: PSA, VSA, TSA, and PTSA. Initially, we performed GCMC simulations for all CoRE COFs and a representative set of hypoCOFs to compute the adsorption data for an equimolar CO_2_/CH_4_ mixture and then used this data to train ML models that accurately predict the CO_2_/CH_4_ mixture adsorption data of COFs. The input of the ML models was designed to be structural, chemical, graph-based, and energy-based properties of COFs, where graph-based descriptors of COFs were produced by using molecular fingerprinting methodologies for the first time in the literature. These ML models were then used to predict the CO_2_/CH_4_ adsorption and separation properties of the entire hypoCOF database for which computationally demanding molecular simulations have not been performed due to the very large number of structures. The top COF adsorbents offering the highest adsorbent performance scores (combination of selectivity and working capacity) and high regenerabilities were identified to highlight the key molecular features of materials to achieve high-performance CO_2_/CH_4_ separation. Our computational methodology will significantly accelerate the discovery of COF adsorbents for natural gas purification by quickly evaluating the CO_2_/CH_4_ separation potential of any new COF bypassing the need for time-consuming molecular simulations and by revealing the fundamental structural and chemical properties of the most promising adsorbents to catalyze the experimental work in designing new COF materials.

## 2 Computational Details

**Figure 1:**
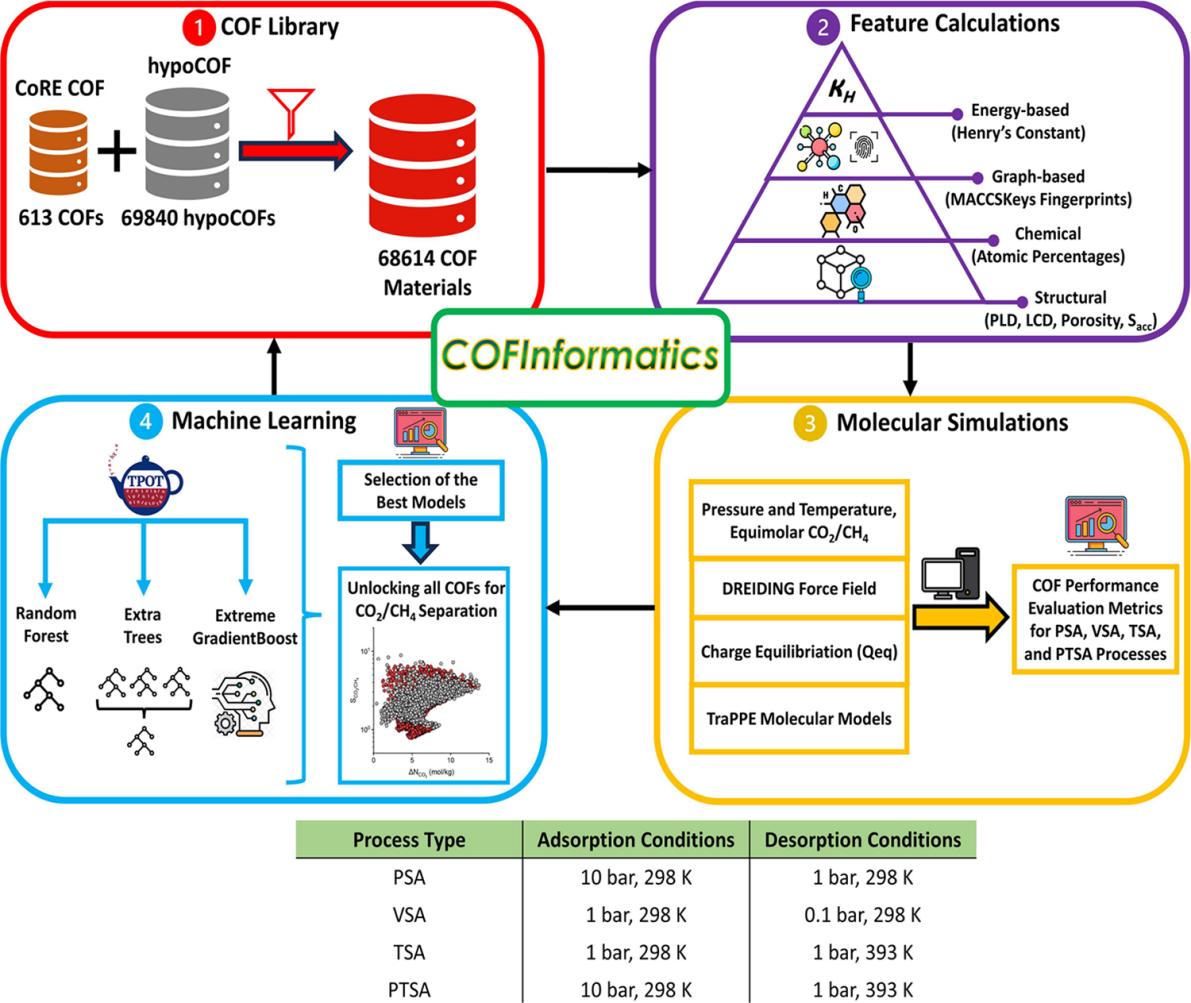
Introduction of our computational approach, COFInformatics: (1) a total of 7340 COFs comprising 543 synthesized COFs from the CoRE COF database and 6797 computer-generated COFs from the hypoCOF database were selected. (2) Structural, chemical, graph-based, and energy-based descriptors were produced for 7340 COFs to be used in the training of ML models. (3) GCMC simulations were performed for these 7340 COFs to calculate their equimolar CO_2_/CH_4_ mixture uptakes, which were then used to calculate the COF adsorbents’ performance evaluation metrics for PSA, VSA, TSA, and PTSA processes. (4) The ML models were developed using different algorithms to accurately predict CO_2_/CH_4_ mixture adsorption data, and the best models with corresponding descriptor groups were selected. The models were retrained with the addition of 200 COFs to the training set to calculate adsorption data of the entire COF library composed of 61,074 unseen and 7540 seen materials for unlocking their CO_2_/CH_4_ separation performance for four different separation processes.

Figure 1 presents an overview of our computational approach, which integrates molecular simulations with ML to analyze the gas adsorption properties of the COFs and hypoCOFs. Our study concentrates on the CoRE COF database (the sixth version)^10^ and the most recent edition of the hypoCOF database,^16^ encompassing 613 synthesized COFs and 69,840 hypoCOFs. We calculated the structural properties of every single COF such as pore limiting diameter (PLD), the largest cavity diameter (LCD), accessible surface area (*S*_acc_), and porosity (ϕ) using the Zeo++ software (version 0.3).^37^*S*_acc_ values were calculated by using a probe radius of 1.82 Å (representing N_2_). Materials with PLDs under 3.8 Å and *S*_acc_ values of zero were excluded to ensure the adsorption of both CO_2_ and CH_4_ in the pores of the COFs. We have intentionally excluded CoRE COFs that contain metals to ensure the uniformity with the hypoCOF data set, which consists entirely of structures free from metals. This approach led to the selection of 543 CoRE COFs and 69828 hypoCOFs for further evaluation.

In the next step, we employed the Molecular Access Keys (MACCSKeys) substructure search method, using the RDKit tool,^38^ to graphically analyze the COF structures for the first time in the literature. Our goal was to conduct an in-depth chemical analysis to identify the key chemical components determining the performance of the CO_2_/CH_4_ separation of the COFs. MACCSKeys uses a predefined set of 167 substructures, represented in Simplified Molecular Input Line Entry System (SMILES) Arbitrary Target Specification (SMARTS) notation, and was recently applied to analyze metal–organic frameworks (MOFs).^39,40^ These substructures were denoted as different “bits” for different chemical groups. For example, bit 163 has a SMARTS notation of “*1∼*∼*∼*∼*∼*∼1” meaning that there is a six-carbon ring in the framework. For each COF, we generated a binary string of “0” and “1”, indicating the absence and presence, respectively, of these bits defined in the MACCSKeys library. We successfully extracted the fingerprint data for 68,614 out of 70,371 total COFs, including both experimental and hypothetical types, and focused our subsequent analyses on this set of materials.

Using the RASPA software,^41^ we studied COFs at industrially relevant pressure and temperature conditions for adsorption-based separation processes, specifically pressures of 0.1, 1, and 10 bar and temperatures of 298 and 393 K, to represent landfill gas separation at PSA, VSA, TSA, and PTSA conditions, as shown in Figure 1.^42−46^ GCMC simulations were performed for all 543 CORE COFs and a representative set of 6797 hypoCOFs, which was selected by sorting the hypothetical materials based on their LCDs, from the smallest to the largest, and then selecting every 10th material. We modeled COF-gas and gas–gas interactions using Lennard-Jones 12–6 and Coulombic potentials and used the DREIDING force field^47^ for the framework atoms. For CO_2_ and CH_4_, we used the TraPPE potentials.^48,49^ The charge equilibration method (*Q*_eq_),^50^ integrated into the RASPA, was used to assign the partial point charges of COFs’ atoms. Electrostatic interactions between CO_2_ molecules and COFs were defined using the Ewald summation.^51^ The reliability of these simulation settings was previously confirmed by showing the good agreement between simulated and experimental adsorption data for single-component CO_2_ and CH_4_ in several studies of COFs.^12,25,26,28^

Our GCMC simulations included 10,000 cycles for setup and 20,000 cycles to compute the average values. Additionally, we computed the heat of adsorption for CO_2_ and CH_4_ gases at infinite dilution and Henry’s coefficients using the Widom insertion technique.^52^ From the GCMC results, we calculated the key adsorbent performance evaluation metrics for COFs; selectivity (*S*_CO_2_/CH_4__), working capacity (Δ*N*_CO_2__), adsorbent performance score (APS), and percent regenerability (*R*%), as detailed in Table S1 in the Supporting Information. APS is the multiplication of selectivity and working capacity, and high *R*% indicates the material’s potential for efficient cyclical use in a separation process. To identify the most promising adsorbents, we ranked all COFs having an *R*% > 85% and pinpointed the top 10 COF adsorbents from both CoRE COFs and hypoCOFs with the highest APSs.

Given the extensive size of the hypoCOF database, which includes 69,840 materials, performing exhaustive molecular simulations on each material would be resource-intensive. To circumvent this, we constructed different ML models using four different feature groups to predict CO_2_/CH_4_ mixture adsorption data of COFs. To develop ML models having high prediction accuracy, our initial step involved analyzing the relationships between the materials’ descriptors. We identified 144 distinct descriptors for 7340 COFs, categorizing them into four groups, as detailed in Table S2. The groups comprise four structural descriptors (PLD, LCD, *S*_acc_, and ϕ), seven chemical descriptors (percentages of C, H, N, O, halogens, nonmetals, and metalloids in COFs), and an energy-based descriptor (Henry’s coefficients for CO_2_ and CH_4_). We calculated the Pearson correlation coefficients to assess the interdependencies among these features, and these correlations were depicted in the correlation matrix given in Figure S1. We also added the information on 132 out of 166 available MACCSKeys molecular fingerprinting bits as graph-based descriptors, consisting of “0” and “1”, since 34 of the predefined substructures in MACCSKeys have not been observed in our COF material space. We first developed ML models encompassing purely structural properties, then we consecutively added chemical, graph-based, and finally energy-based descriptors as features. To train the ML models, we utilized the simulated gas adsorption data of 7340 materials comprising both CoRE COFs and hypoCOFs as the target variable and descriptors as the input as detailed in Table S2. As a result, we developed 16 different ML models to predict mixture CO_2_ uptakes at 0.1 bar-298 K, 1 bar-298 K, 1 bar-393 K, 10 bar-298 K, and 8 different ML models to predict mixture CH_4_ uptakes at 1 bar-298 K, 10 bar-298 K.

The tree-based pipeline optimization tool (TPOT)^53^ within the auto ML framework was employed to determine the most suitable algorithms and to fine-tune their hyperparameters. We used the regression algorithms from the scikit-learn^54^ library for the model selection. A stratified sampling approach was adapted to maintain a consistent feature distribution across the training and test data sets, allocating 80% of the data for training and 20% for testing. To avoid overfitting, we implemented 5-fold cross-validation. The effectiveness of the ML models was assessed using several statistical accuracy metrics; the coefficient of determination (*R*^2^), mean absolute error (MAE), root-mean square error (RMSE), and Spearman’s ranking correlation coefficient (SRCC), as reported in Table S3. Based on these metrics, as shown in Tables S4–S7, a variety of regression models, including the Extra Tree,^55^ GradientBoost,^56^ and XG-Boost,^57^ were selected for their ability to accurately predict CO_2_/CH_4_ mixture uptakes, which will be explored in more detail in subsequent sections. All of the energetic descriptors together with other structural, chemical, and graph-based ones made available with our models for COFs in our depository https://github.com/gokhanonderaksu/COFS_CO2CH4_ML.

In the final phase, we tested the adaptability of our ML models by using them to predict the CO_2_/CH_4_ mixture adsorption data of a distinct subset comprising randomly selected unseen 19050 hypoCOFs under specified conditions of PSA, VSA, TSA, and PTSA processes using the ML models. For these unseen hypoCOFs, we also performed GCMC simulations and compared the ML-predicted mixture uptakes with the simulated ones to further validate the transferability of our models in terms of accurately predicting the unseen data. Finally, we utilized the ML models for the entire COF library composed of 68,614 COFs to predict their CO_2_/CH_4_ adsorption and separation properties for four different processes. We identified the top-performing materials exhibiting the highest APSs and then performed a detailed molecular-level analysis to unlock the structural and chemical descriptors of COFs that lead to superior CO_2_/CH_4_ separation performance.

## 3 Results and Discussion

**Figure 2:**
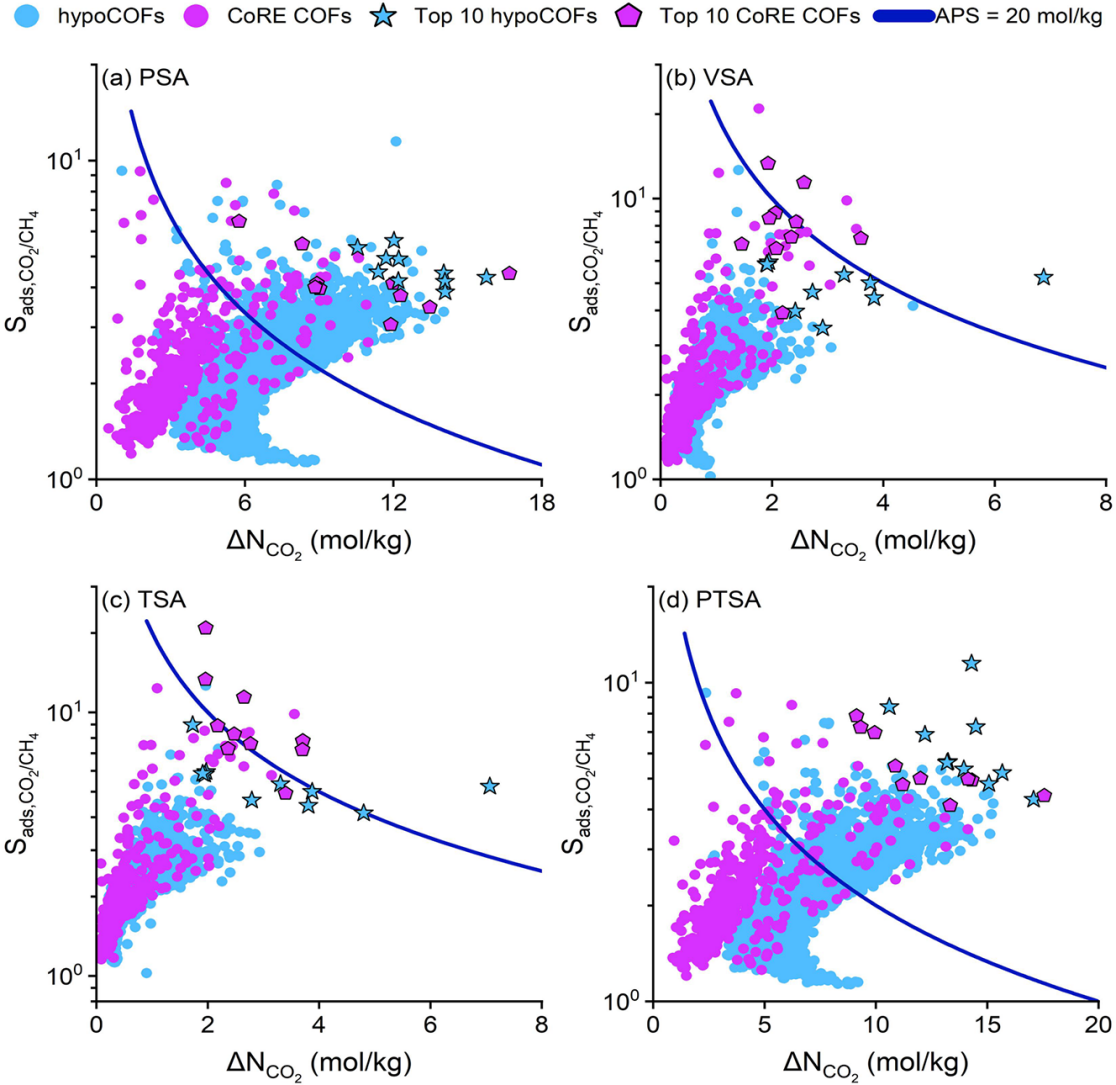
Simulated selectivities, working capacities, and adsorbent performance scores of 543 CoRE COFs and 6797 hypoCOFs for the CO_2_/CH_4_:50/50 mixture separation under (a) PSA, (b) VSA, (c) TSA, and (d) PTSA conditions. Stars and pentagons represent the top 10 hypoCOFs and CoRE COFs, respectively, showing *R*% > 85% with the highest APSs.

Based on the adsorption data obtained from molecular simulations for 543 CoRE COFs and 6797 hypoCOFs, we computed CO_2_/CH_4_ selectivities and CO_2_ working capacities of every material for CO_2_/CH_4_:50/50 mixture separation under four different process conditions as shown in Figure 2. The ideal adsorbents are desired to offer both high selectivities and high working capacities. Therefore, we calculated and used the APSs of the COFs as the key performance metric to identify the most promising adsorbents. Almost all COF and hypoCOF adsorbents were found to be CO_2_ selective since CO_2_ has additional electrostatic interactions with the COFs which is absent in the case of CH_4_. Figure 2a,b shows that CO_2_/CH_4_ selectivities and CO_2_ working capacities of CoRE COFs range between 1.2 and 9.3 (1.2–20.9) and 0.5–16.7 (0.1–3.6) mol/kg under PSA (VSA) conditions, respectively. For hypoCOFs, the selectivities and working capacities were calculated to be similar, in between 1.1 and 11.5 (1.0–12.6) and 1.0–15.8 (0.3–6.9) mol/kg under PSA (VSA) condition. 59 CoRE COFs have high APSs (>20 mol/kg) at PSA conditions, while under VSA condition, only 9 COFs achieved high APSs. For hypoCOFs, 680 structures surpassed the APS = 20 mol/kg limit at the PSA condition, whereas only a single COF was above the limit at the VSA condition. These results suggest that COFs offer higher working capacities under PSA condition, whereas slightly higher selectivities were observed for the VSA process. Figure 2c shows that both synthesized and hypothetical COFs have slightly higher working capacities (0.1–7.1 mol/kg) under TSA condition compared to VSA, which leads to more COF adsorbents (12) with high APSs (>20 mol/kg) compared to the number of COFs found at VSA condition (10). The combination of pressure and temperature swing leads to higher CO_2_ working capacities (0.9–17.6 mol/kg) and increases the total number of CoRE COFs (76) and hypoCOFs (813) achieving high APSs (>20 mol/kg) compared to those identified for other processes as shown in Figure 2d. All in all, both synthesized and hypoCOFs achieve the highest APSs under PTSA condition thanks to very high CO_2_ working capacities.

Our previous work showed that many COFs offering high APSs can suffer from low regenerabilities in CO_2_/H_2_ and CH_4_/H_2_ separations due to the strong confinement of CO_2_ molecules in the pores and hence difficulty of desorption.^28,36^Figures S2a,b and S3a,b show the relationship between APS and *R*% for PSA and VSA processes, and COFs achieving high APSs (>20 mol/kg) suffer from low *R*% (<85%). For the TSA process, however, a different trend was observed such that COFs with high APSs also have high *R*%, as shown in Figures S2c and S3c. However, the number of both CoRE COFs and hypoCOFs achieving high *R*% (>85%) is limited at TSA condition compared to PSA and VSA conditions. This is due to higher CO_2_ uptakes observed at the desorption condition of TSA (1 bar-393 K) compared to the ones acquired at the desorption condition of the VSA process (0.1 bar-298 K). This results in lower CO_2_ working capacities and regenerabilities for most COFs at TSA conditions, showing that changing the pressure is more effective than changing the temperature for desorption. Specifically, 442 (514) CoRE COFs and 6504 (6705) hypoCOFs demonstrated *R*% values exceeding 85% under PSA (VSA) conditions. Under TSA condition, only 46 CoRE COFs and 79 hypoCOFs can pass *R*% = 85% limit, proving the inefficiency of only temperature swing approach. In Figures S2d and S3d, where both pressure and temperature swing were considered for the PTSA process, except for only one hypoCOF, all COFs surpass *R*% = 85% limit and achieve very high *R*% (>95%). Considering these results, PTSA was considered as the most efficient process for COF adsorbents to separate the CO_2_/CH_4_ mixture.

We then identified the top 10 COFs from both CoRE COFs and hypoCOFs with the highest APSs while maintaining an *R*% above 85% only for the PTSA process. The top hypoCOFs achieve much higher APSs (up to 164.2 mol/kg) compared to the top CoRE COFs (up to 77.6 mol/kg) identified for the PTSA process. The details of these top COFs under PTSA condition, including their structural properties and calculated performance metrics, are listed in Tables S8 and S9. When we compared structural and performance properties of both material spectrum, we observed that hypoCOFs have a much wider range for pore size (PLD: 3.7–85.4 Å and LCD: 4.0–87.0 Å) compared to CoRE COFs (PLD: 1.3–60.7 Å and LCD: 2.6–60.8 Å), and the top-performing hypoCOFs generally comprise narrow pores (PLD: 3.8–6.4 Å and LCD: 7–12 Å).

The next step was to construct ML models capable of swiftly estimating the CO_2_/CH_4_ mixture adsorption and separation performance of the entire hypoCOF database, which includes a substantial number of materials (∼68,000 and potentially more that might be added in the future). To achieve this, we trained our ML models for 7340 COFs, 543 CoRE COFs, and 6797 hypoCOFs, first by only using structural descriptors (red), then adding the chemical descriptors (blue), graph-based descriptors (green), and energy-based descriptors (magenta) consecutively. As a result, 24 regression models were developed to predict the CO_2_/CH_4_ mixture uptakes of all COFs at predefined process conditions. Comprehensive details for the type of each regression model together and their optimized hyperparameters are presented in Tables S4–S7. We evaluated the prediction accuracies of these models by comparing the ML-predicted CO_2_ and CH_4_ adsorption data with the results of molecular simulations in Figures S4–S7 based on the statistical accuracy metrics; *R*^2^, MAE, RMSE, and SRCC, which were calculated for each model.

**Figure 3:**
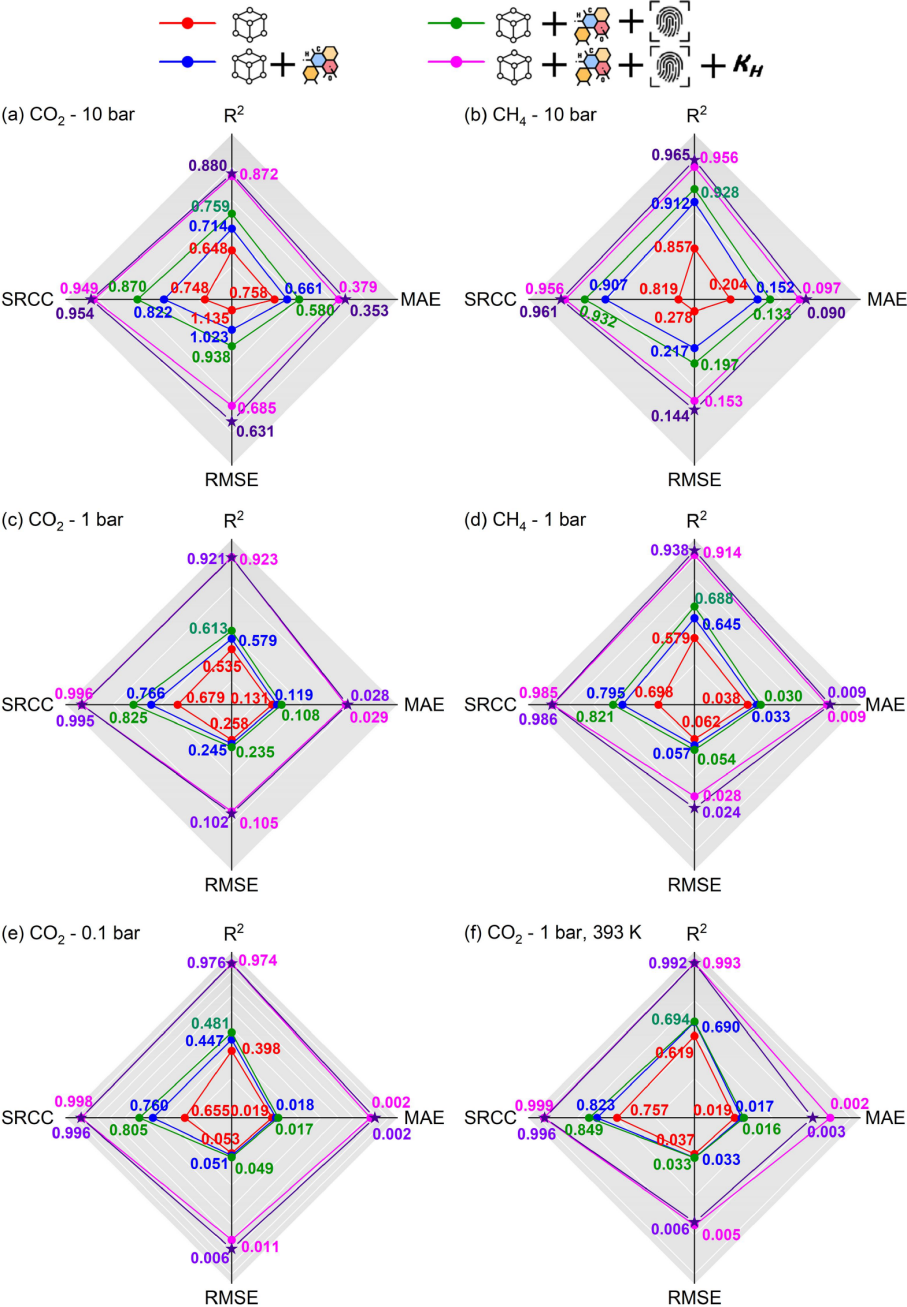
ML model evaluation metrics; *R*^2^, MAE, RMSE, and SRCC calculated for mixture CO_2_ and CH_4_ uptakes of hypoCOFs in the test set at (a,b) 10 bar, (c,d) 1 bar, (e) 0.1 bar-298 K, and (f) for CO_2_ uptakes at 1 bar-393 K. Data axes for *R*^2^ and SRCC are in ascending order, while for MAE and RMSE, axes are in the descending order. Trained ML models were developed using only structural descriptors (red); both structural and chemical descriptors (blue); structural, chemical, and graph-based descriptors (green); structural, chemical, graph-, and energy-based descriptors (magenta); and “retrained” models were developed using all descriptors (purple and star).

Figure 3 shows a comparison of these accuracy metrics for our test set constructed with different feature groups. The best models are expected to achieve both high *R*^2^ and SRCC with low MAE and RMSE. Figure 3a–f shows that ML models with all-inclusive descriptors (depicted as magenta) outperform other models, regardless of the gas type or adsorption condition. For example, Figure 3a shows that the ML model constructed only with structural descriptors has an *R*^2^ and MAE of 0.648 and 0.758, respectively, for the test set. The addition of both chemical and graph-based descriptors increases the *R*^2^ value to 0.759 and decreases the MAE value to 0.580. Finally, the addition of energy-based descriptor, Henry’s constant of CO_2_, provided an almost 10% increase in *R*^2^ (0.872) and a decrease in MAE (0.379). Combining structural, chemical, graph-, and energy-based features led to the most accurate ML models.

We then evaluated the CO_2_/CH_4_ separation capabilities of these 19050 unseen hypoCOFs by comparing their ML-predicted and simulated gas uptakes. CO_2_ uptakes of unseen hypoCOFs were accurately predicted at all conditions (*R*^2^ > 0.8) as shown in Figure S8a, c, e, and f. For CH_4_ uptakes, although ML predictions were good at 1 bar-298 K (Figure S8d), the ML model failed to predict CH_4_ uptakes at 10 bar-298 K (Figure S8b). In supervised learning, regressor models have limitations to make accurate predictions for data points that are not included in their training set.^58^ Although our training set includes a range of CH_4_ uptake values (0.29–8.40 mol/kg) that cover those in the unseen data (1.52–6.12 mol/kg) at 10 bar-298 K, the model underestimated simulated results. This underestimation may arise because the number of materials in the training set with high CH_4_ uptake (>6 mol/kg) is relatively few (only 32 COFs) compared to the trained data set composed of 7340 COFs. Thus, we inferred that the model may not learn enough about the materials having high CH_4_ uptakes to make accurate predictions for similar cases outside of its training set.

To solve this problem, we retrained the ML models using an additional 200 hypoCOFs having the highest CH_4_ uptakes among 19050 unseen hypoCOFs. Table S8 summarizes the ML models retrained with 7550 seen data and their hyperparameters, and Figure S9 shows their statistical accuracies. We observed that retrained ML models, for which data are shown as purple stars in Figure 3, demonstrated comparable accuracies to the initial ML models in terms of predicting CO_2_ and CH_4_ uptakes of seen COFs. For CO_2_ uptakes at 10 bar-298 K, 1 bar-298 K, 0.1 bar-298 K, and 1 bar-393 K conditions, *R*^2^ values of retrained ML models were in between 0.880 and 0.992 for the test set, very similar to those calculated for initial ML models (0.872–0.993), as shown in Figure 3a, c, e, and f.

Figure 3b,d shows slight improvements in predicting CH_4_ uptakes of test set at both 10 bar-298 K and 1 bar-298 K, as *R*^2^ values increased from 0.956 to 0.965 and 0.914 to 0.938, respectively. We then performed the unseen data analysis for the remaining 18850 hypoCOFs using the retrained ML models. Figure S10a, c, e, and f shows that our retrained models predict CO_2_ uptakes of unseen hypoCOFs (*R*^2^: 0.860–0.995) at the corresponding conditions as accurate as original models (*R*^2^: 0.813–0.988). Figure S10b represents that the retrained ML model successfully predicts unseen CH_4_ uptakes at 10 bar-298 K compared to the initial model, as the *R*^2^ value increased from 0.369 to 0.700 and the MAE decreased from 0.282 to 0.180, solving the problem. Figure S10d shows a similar improvement of the retrained model for CH_4_ uptakes at 1 bar-298 K (*R*^2^: 0.951) compared to earlier (*R*^2^: 0.947). Thus, we concluded that the retrained models can precisely predict the CO_2_ and CH_4_ uptakes of 7540 seen COFs and 18850 unseen COFs.

**Figure 4:**
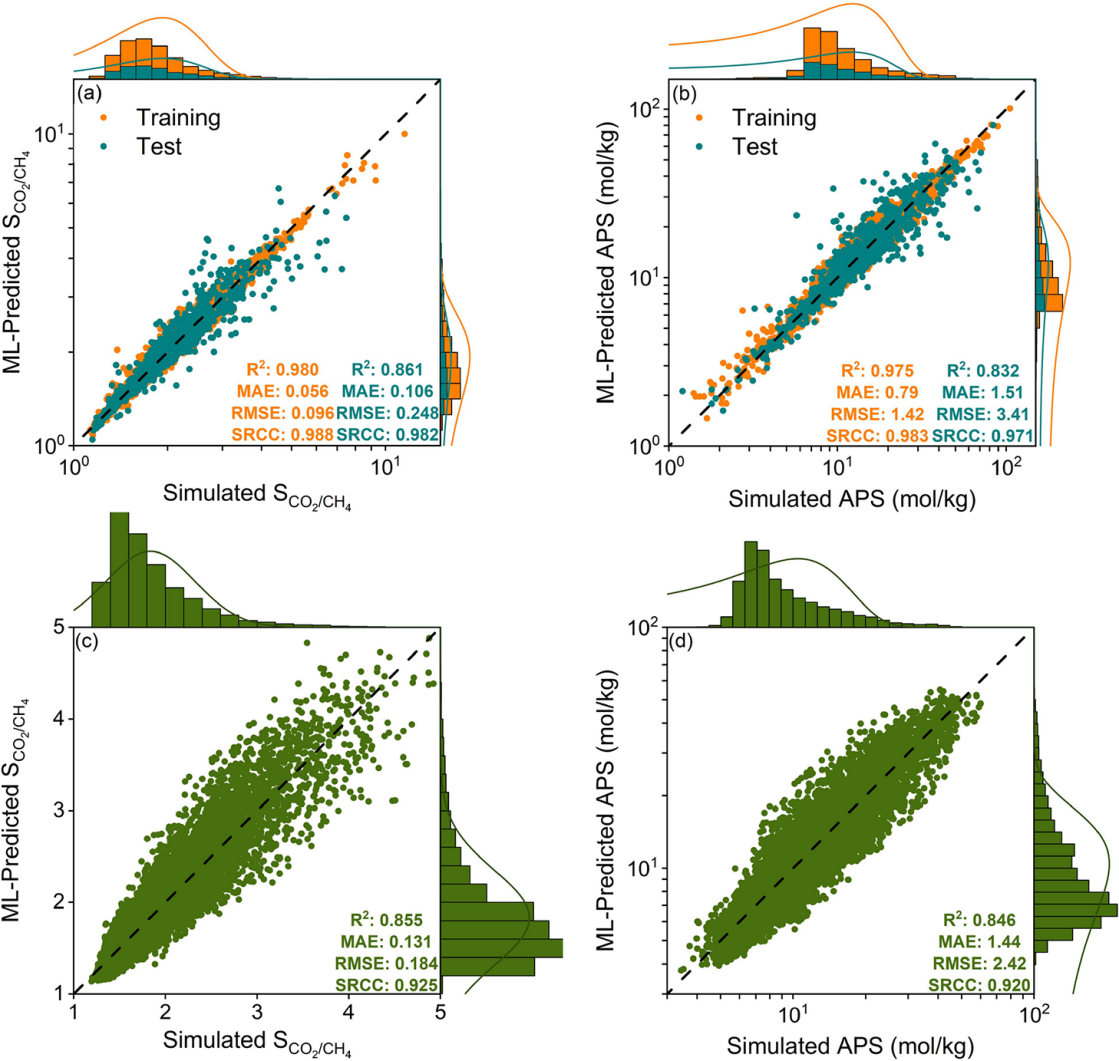
Comparison of ML-predicted and simulated selectivities and APSs of (a, b) 7540 seen COFs and (c, d) 18850 unseen hypoCOFs under the PTSA condition.

We then applied these retrained models to determine CO_2_/CH_4_ selectivities, working capacities, and APSs of COFs. Figure 4a,b displays the agreement between ML-predicted and simulated selectivity and APS for 7540 COFs under the most optimal PTSA process together with their normal distributions. Both simulated and ML-predicted selectivities and APSs have very similar distributions in the training and test sets, signifying that our models can reflect well what is trained on the corresponding test sets. The ML-predicted selectivities and APSs show strong correlation with the simulation data with *R*^2^ values for test sets being 0.861 and 0.832, respectively. Simulated selectivity and APS for the top 10 CoRE COFs were calculated to be between 4.1 and 7.9 and 53.6–77.6 mol/kg, while ML-predicted values were in between 3.4 and 7.1 and 25.8–69.1 mol/kg, showing the accuracy of models. Simulated (ML-predicted) selectivities and APSs for the top hypoCOFs were computed to be between 4.3 and 11.5 (4.1–10) and 72.6–164.2 (69.1–133.9) mol/kg. An important result is that our ML models found 8 (10) of the top 10 CoRE COFs (hypoCOFs) identified from the simulation results. This rapid ML prediction capability, which takes mere seconds as opposed to the several weeks required for molecular simulations, is very beneficial for efficiently pinpointing the most promising COFs among thousands of candidate materials.

We evaluated the CO_2_/CH_4_ separation capabilities of these 18,850 unseen hypoCOFs by comparing their selectivity and APS calculated from ML-predicted and simulated gas uptakes under the optimal PTSA conditions. ML-predicted selectivities ranged from 1.1 to 4.9, while the simulated values varied from 1.1 to 5.4 as shown in Figure 4c. Around 90% of both the ML-predicted and simulated selectivities are predominantly within the range of 1–3. Additionally, high *R*^2^ and low error (MAE, RMSE) values indicate the accuracy of our models in predicting the CO_2_/CH_4_ selectivities for unseen data. A similar pattern was observed for APS where ML-predicted and simulated values were found to be 3.8 to 55.1 mol/kg and 3.4 to 60 mol/kg, respectively, as shown in Figure 4d. The majority (over 90%) of both ML-predicted and simulated APSs cluster in the 6–25 mol/kg range, with similar distribution patterns. The models’ accuracy was further confirmed by the high *R*^2^ and SRCC values (0.846 and 0.920), validating their reliability in ranking materials.

We demonstrated the accuracy of our predictions for unseen COFs regarding both CH_4_ and CO_2_ uptakes in Figure S10 and showed that the predicted uptakes for unseen COFs fell within the range of uptakes taught in the training set. The ML-predicted selectivities of unseen COFs (1.1–4.9) were also within this range, albeit slightly lower compared to those in the training set (1.1–10) at 10 bar and 298 K, and those are the natural consequences of the extrapolation limitation of supervised algorithms. These findings demonstrate the models’ applicability in rapidly and accurately evaluating materials not included in the training set and highlight the models’ potential for unlocking the CO_2_/CH_4_ separation performance of the entire COF spectrum.

**Figure 5:**
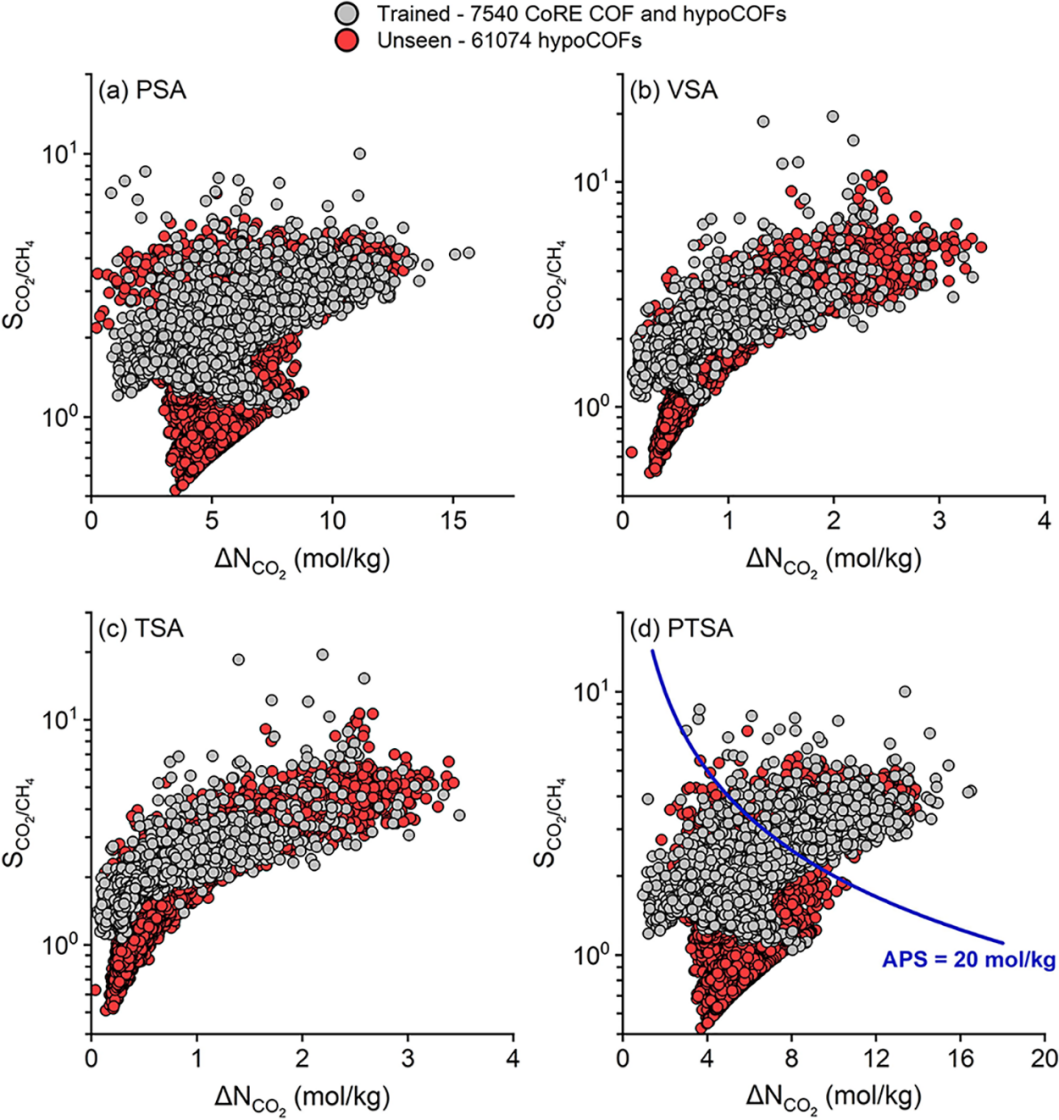
ML-predicted CO_2_/CH_4_ adsorption performance of the whole COF material spectrum composed of 68,614 structures for (a) PSA, (b) VSA, (c) TSA, and (d) PTSA processes.

In the final phase of our work, we applied our ML models to evaluate the CO_2_/CH_4_ selectivity and CO_2_ working capacity of all 68,614 structures available in the COF space. Figure 5 represents the most comprehensive analysis of CO_2_/CH_4_ separation capabilities across the largest spectrum of COFs under four different adsorption-based separation conditions, a first in the literature. For 61074 unseen hypoCOFs, ML-predicted CO_2_/CH_4_ selectivities and CO_2_ working capacities change in between 0.4 and 7.1 (0.5–10.7) and 0.2–13.0 mol/kg (0.1–3.4) mol/kg under PSA (VSA) condition, respectively, as shown in Figure 5a,b. For the TSA process, CO_2_ working capacities range between 0.04 and 3.4 mol/kg as shown in Figure 5c. Figure 5d shows that at the PTSA process, CO_2_/CH_4_ selectivities and CO_2_ working capacities of hypoCOFs were predicted to be 0.4–7.1 and 1.6–13.9 mol/kg, respectively, which are the highest values among the four processes we considered in this work. Overall, we have two key findings: (i) The distribution of performance metrics in both trained and untrained data sets is quite similar, with many high-performing materials emerging from the trained set, especially in the PTSA process. (ii) Under all conditions, we observed that COFs with high CO_2_/CH_4_ selectivities can also achieve high CO_2_ working capacities.

We then focused on the discovery of new materials achieving high APSs (>20 mol/kg) at the PTSA condition to see if there is a new material outperforming the top materials that we identified based on the results of molecular simulations of CoRE COFs and a subset of hypoCOFs that we examined in the beginning of our work. According to our molecular simulation results, 76 out of 543 CoRE COFs and 890 out of 6997 hypoCOFs surpassed the APS = 20 mol/kg limit in the training data set. In the unseen data set, ML results in Figure 5d show that there are 6056 hypoCOFs offering high APS > 20 mol/kg. Moreover, for some materials, the CO_2_ uptake was predicted to be lower than the CH_4_ uptake, resulting in 6176 (8956) materials with CO_2_ selectivity values slightly less than 1, 0.4–1 (0.5–1) under conditions of 10 (1) bar and 298 K. This is due to slight underpredictions (overpredictions) in CO_2_ (CH_4_) uptakes by the models for these materials. Although these under- and overpredictions exist within our training set, they are within a range, according to our calculated RMSE and MAE values, that does not significantly affect the accuracy. Therefore, such results are feasible within ML predictions; however, the focal point here should be that ML models marked these materials to be unpromising for CO_2_/CH_4_ separation under the specified conditions. These findings indicate that ML models enable the rapid identification of optimal COF adsorbents for CO_2_/CH_4_ separation from a large COF material space.

So far, we have concentrated on the structural characteristics of the top-performing COFs and concluded that they tend to have narrow-pored structures. We now focused on the chemical characteristics of the top-performing materials to have better molecular insights about the effect of chemical environments of COFs on their CO_2_/CH_4_ separation performances. We used the bits obtained from the MACCSKeys fingerprinting analysis and listed the very common and unique bits observed for the top 10 materials in Table S11. A total of 93 distinct bit types were observed among the top 10 COFs, while 8 specific bits (105, 131, 141, 145, 149, 160, 163, and 165) were seen in all of them. For instance, bit 131 is indicative of an atom, other than carbon or hydrogen, bonded to at least one hydrogen atom, while bit 160 indicates carbon atoms bonded to three or four hydrogen atoms. These specific bits are prevalent in the hypoCOF material spectrum, forming the fundamental elements, “skeleton” of the COFs.

**Figure 6:**
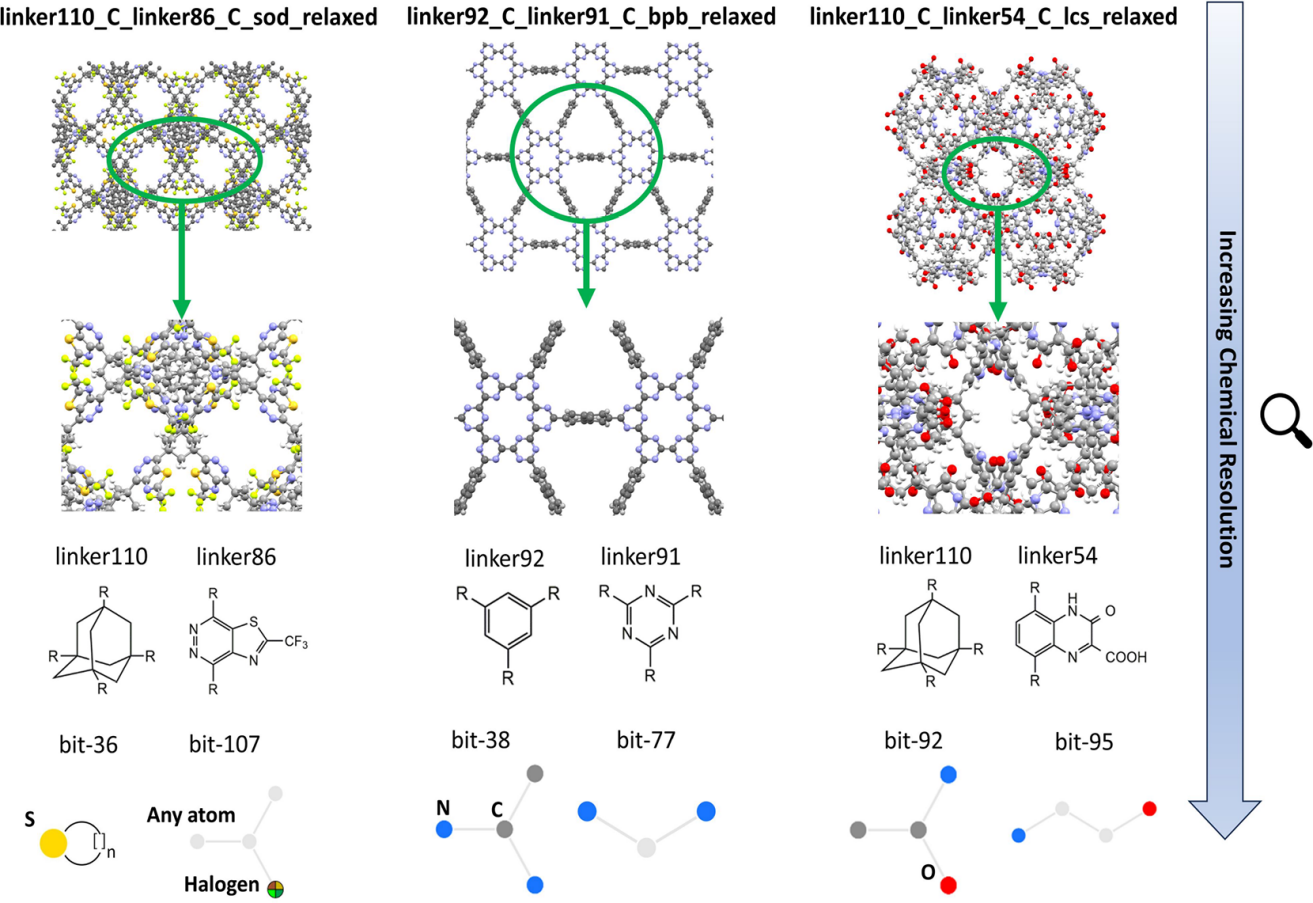
Detailed chemical analysis of the top three hypoCOFs identified for CO_2_/CH_4_ separation under PTSA condition. The corresponding depictions of linker subunits and unique bits were given. For bit representations, yellow, blue, red, and dark gray spheres represent S, N, O, and C, while light gray and mixed-color signify “any atom” and “one of the halogen atoms”, respectively.

The top three most promising hypoCOFs with highest APS and *R*% > 85% are linker110_C_linker86_C_sod_relaxed, linker92_C_linker91_C_bpb_relaxed, and linker110_C_linker54_C_lcs_relaxed, as depicted in Figure 6. These hypoCOFs include linker units such as linker110 (adamantane-based), linker86 (fluorine functionalized), linker91 (triazine-based), linker92 (benzene-based), and linker54 (carboxylic acid functionalized) known for their high CO_2_ affinity as discussed in previous studies.^15,28^ For the linker110_C_linker86_C_sod_relaxed structure, unique bits such as bit-36 (sulfur heterocyclic units) and bit-42 (fluorine group), specific to linker86, were identified. These two bits appear in 367 and 124 materials in the hypoCOF space, respectively. Such halogen groups are well-known for favoring the adsorption of CO_2_ molecules inside the pores of COFs, resulting in high selectivities.^12,28^ Unique N-containing 6-ring, triazine structures were identified in the case of the linker92_C_linker91_C_bpb_relaxed structure through bits such as bit-38 (C–N–C branching) and bit-77 (two bonded N atoms), which are present in many structures, 14236 and 17144 hypoCOFs, respectively. Similar types of COFs were previously identified as the top materials for CO_2_ capture applications owing to their triazine and aromatic subunits which are favorable CO_2_ adsorption sites as well.^15,59^ For the linker110_C_linker54_C_lcs_relaxed structure, unique bits like bit-89 (5-atom chain capped with O atoms), bit-92 (tetrahedral C bonding with N, C, and O atoms), and bit-95 (4-atom chain capped with N and O atoms) were observed and found in a very large number of hypoCOFs, 4830, 12,449, and 16,786 materials, respectively. COFs functionalized with carboxylic groups may be promising candidates for CO_2_ capture and separation applications.^60,61^ Overall, our analysis shows that specific chemical environments, such as the presence of halogen, carboxylic acid, triazine, and benzene groups in COFs, significantly enhance their CO_2_/CH_4_ separation performance, providing valuable insights for designing new COFs in future research.

Finally, we compared the results obtained through molecular simulations and ML predictions to those reported experimentally for COFs. The CO_2_/CH_4_ selectivity of the synthesized COFs was reported to vary between 1.3 and 12 using the Ideal Adsorbed Solution Theory (IAST) at 1 bar, with temperatures ranging from 273 to 298 K in different experimental studies.^62−66^ The CO_2_/CH_4_ selectivities of trained COFs that we obtained from molecular simulations (1.2–20.9) and ML models (1.1–19.5) are consistent with these experimentally reported mixture selectivities at 1 bar and 298 K, underscoring the precision of our computational methodology. We also compared the CO_2_/CH_4_ separation performances of COFs and hypoCOFs studied in this work to those of zeolites and MOFs reported in the literature. The highest reported CO_2_/CH_4_ selectivities were 10 and 40 for MOR^67^ and NaX^68^ zeolites, respectively, by molecular simulations at 1 bar, 300 K for separation of an equimolar mixture. Altintas et al. computed CO_2_/CH_4_ selectivities of 3816 MOFs in the range of 1–1.3 × 10^4^ at 1 bar, 298 K for the separation of an equimolar mixture.^69^ In our study, top-performing COFs and hypoCOFs exhibited CO_2_/CH_4_ mixture selectivities reaching up to 19.5 and 15.3 at 1 bar and 298 K, respectively, showing that they are compatible with zeolites but mostly noncompatible to MOFs. We also note that in our previous studies, we examined the impact of presence of water on the adsorption of CO_2_-containing mixtures (CH_4_/C_2_H_6_/CO_2_/C_3_H_8_/H_2_S/H_2_O and CO_2_/N_2_/H_2_O) in synthesized COFs and showed that humidity does not significantly affect the separation performance of hydrophobic COFs, whereas it adversely affects the CO_2_ selectivities of hydrophilic COFs due to the competition between CO_2_ and H_2_O molecules for the available adsorption sites.^25,27^

We have shown the potential of nearly 70,000 hypoCOFs for CO_2_/CH_4_ separation by using ML models and identified the top-performing materials. It is important to note that these materials should be mechanically and chemically stable under a variety of operating conditions to find a place in real applications. COFs stand out by maintaining high crystallinity, porosity like MOFs and zeolites, yet they additionally offer highly stable structures that withstand extreme chemical conditions such as boiling water, strong acids, and bases.^70,71^ They also have very high bulk moduli compared to zeolites, showing their high mechanical stability.^72,73^

## 4 Conclusions

In this study, we introduced COFInformatics, a computational approach combining molecular simulations and ML techniques to evaluate the CO_2_/CH_4_ mixture separation performance of the entire COF spectrum consisting of both synthesized and hypothetical structures. We produced structural, chemical, graph-based (molecular fingerprints), and energy-based descriptors of every single COF and developed ML models that use all these features to accurately predict CO_2_/CH_4_ mixture adsorption and separation properties of all 68,614 COFs under four different separation processes, PSA, VSA, TSA, and PTSA. Results showed that PTSA stands out as the most efficient process for natural gas purification, as COFs achieve high CO_2_/CH_4_ selectivities (up to 10) and CO_2_ working capacities (up to 16.5 mol/kg). The best COF adsorbents identified by COFInformatics were analyzed in detail to reveal which molecular features lead to high-performance materials for CO_2_/CH_4_ mixture separation. COFs having relatively narrow pores (LCD < 15 Å and ϕ < 0.7) and containing halogen or carboxylic acid functional groups, triazine and aromatic benzene subunits were found to strongly favor CO_2_ adsorption, resulting in high CO_2_/CH_4_ selectivities. These chemical insights will act as guidelines to design novel COF adsorbents tailored to the CO_2_/CH_4_ separation. This study aims to significantly widen our understanding of the CO_2_/CH_4_ mixture adsorption and separation performances of COFs by integrating ML and molecular simulations. Our approach rapidly generates data for all COFs, reducing data acquisition time from months to seconds. It also provides a user-friendly option for researchers without computational expertise, replacing complex molecular simulations with accessible models. We believe that COFInformatics introduced in this work will significantly accelerate accurate evaluation of COFs for CO_2_/CH_4_ separation, bypassing the brute force molecular simulations, which require enormous amounts of computational time and resources.

## Supporting Information Available

Correlation matrix between the structural, chemical, and energy-based properties of the trained COF set; *R*%-APS relations of CoRE COFs and hypoCOFs; comparisons of the simulated and ML-predicted CO_2_ and CH_4_ uptakes of trained COFs by models constructed with structural, chemical, graph-based, energy-based descriptors; and comparisons of ML-predicted and simulated CO_2_ and CH_4_ uptakes of unseen hypoCOFs using original and retrained ML models. All ML scripts are available at https://github.com/gokhanonderaksu/COFS_CO2CH4_ML (PDF)
