# Supplementary material for: Rapid and Accurate Screening of the COF Space for Natural Gas Purification: COFInformatics
Source: ACS Appl Mater Interfaces. 2024 Apr 8;16(15):19806–18. doi: 10.1021/acsami.4c01641 (PMC11040585; doi:10.1021/acsami.4c01641)
Supplement: Supplementary file 1 — am4c01641_si_001.pdf [file am4c01641_si_001.pdf]

Supporting Information

for

Rapid and Accurate Screening of the COF Space for Natural Gas Purification: COFInformatics

Gokhan Onder Aksu, Seda Keskin*

Department of Chemical and Biological Engineering, Koc University, Rumelifeneri Yolu, Sariyer, 34450, Istanbul, Turkey

Submitted to *ACS Applied Materials and Interfaces*

*Corresponding author. E-mail: skeskin@ku.edu.tr Phone: +90 (212) 338-1362.

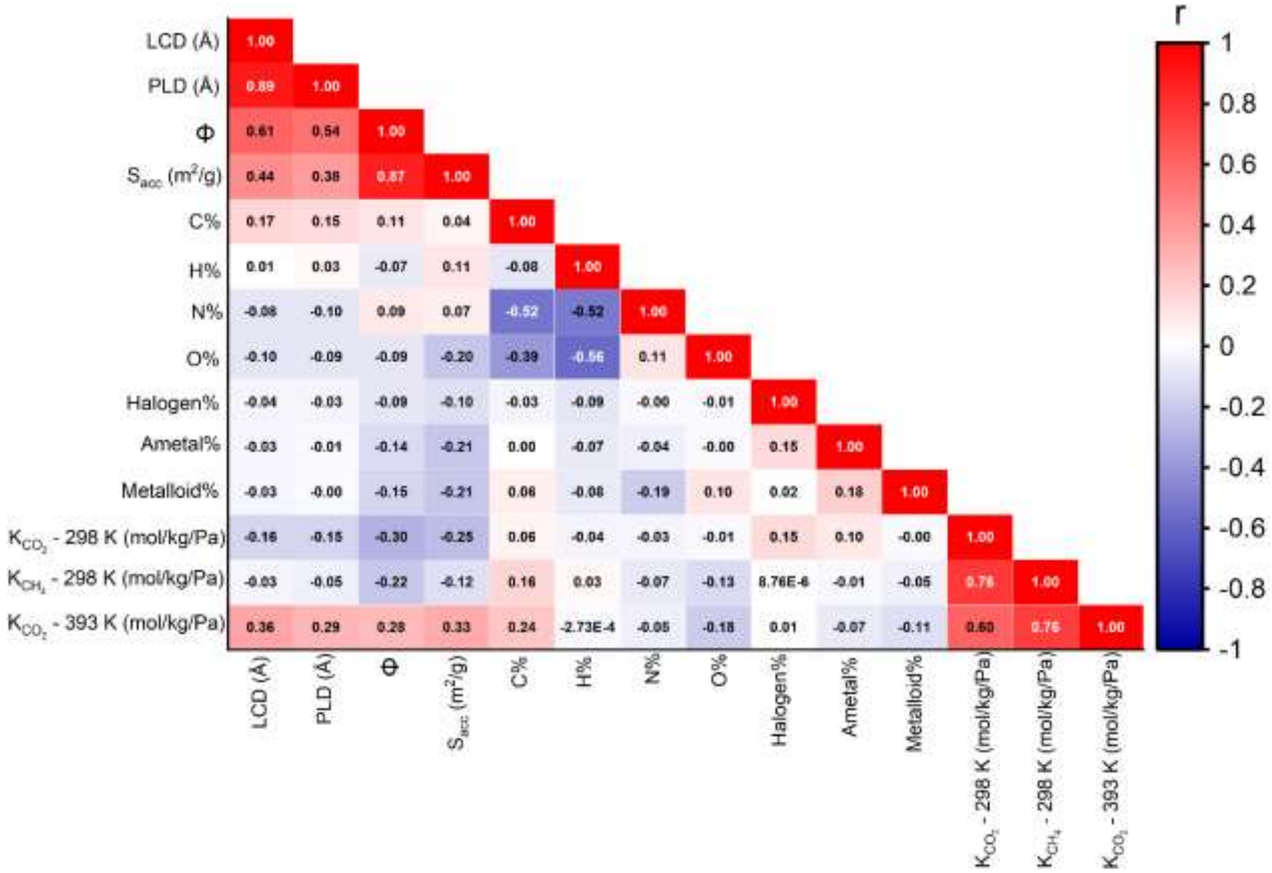

Figure S1. Correlation matrix for structural, chemical, and energy-based descriptors of 7340 COFs comprising of 543 CoRE COFs and 6797 hypoCOFs. Pearson coefficients (r) are provided for the relationship between each descriptor.

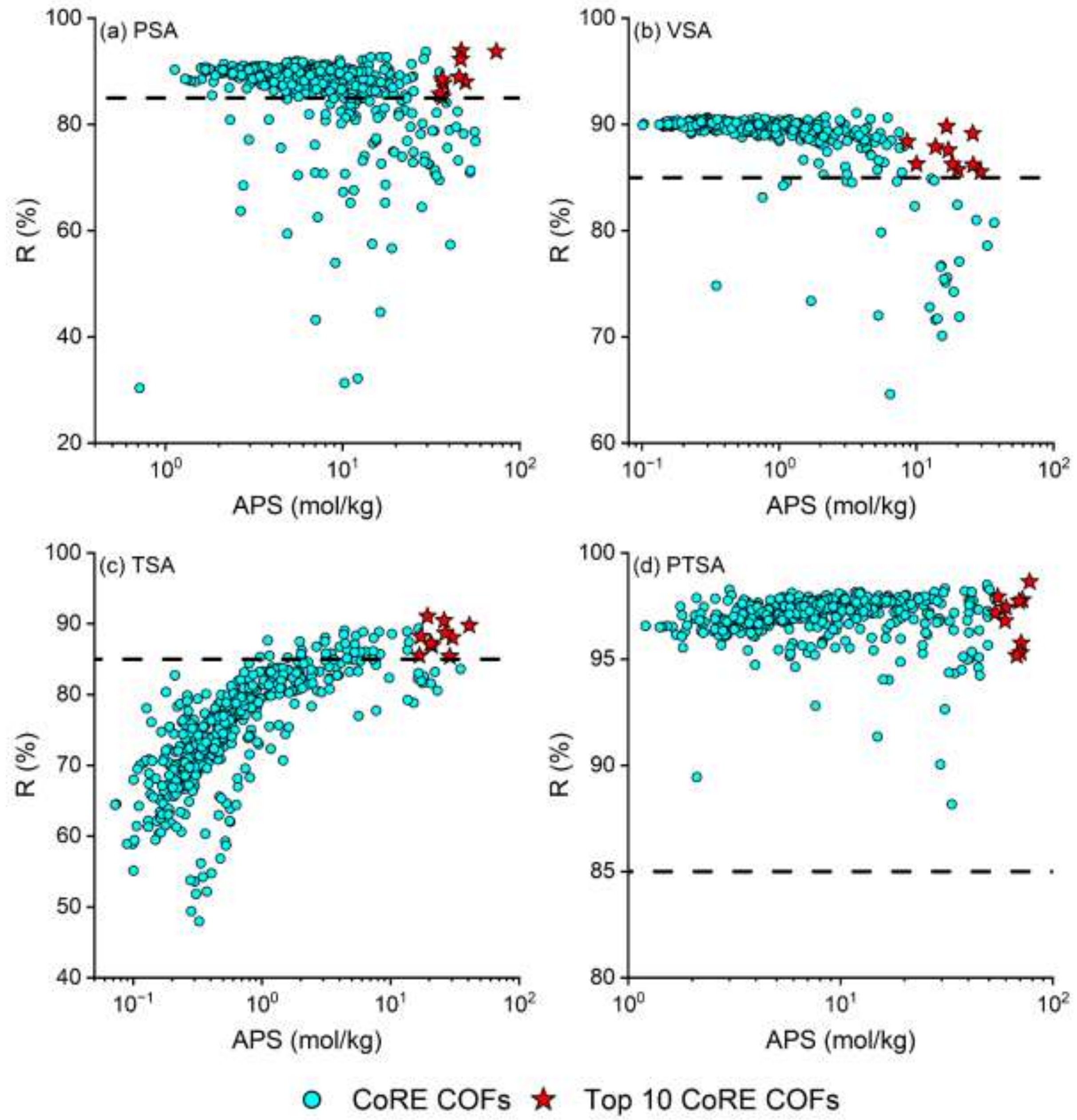

Figure S2. R% and APS of 543 CoRE COFs computed for CO_2/CH_4:50/50 separation at (a) PSA, (b) VSA, (c) TSA, and (d) PTSA conditions.

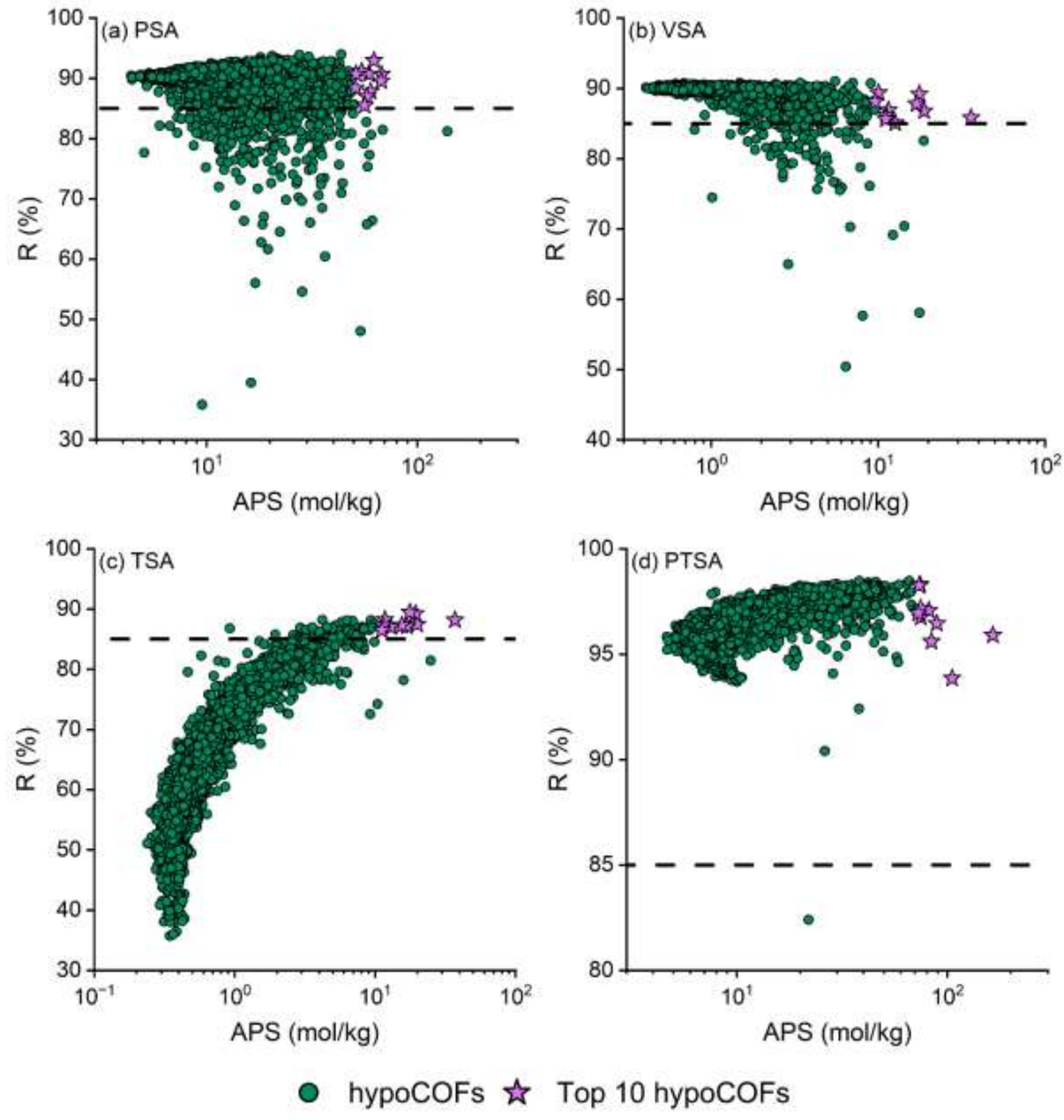

Figure S3. R% and APS of 6797 hypoCOFs computed for CO_2/CH_4:50/50 separation at (a) PSA, (b) VSA, (c) TSA, and (d) PTSA conditions.

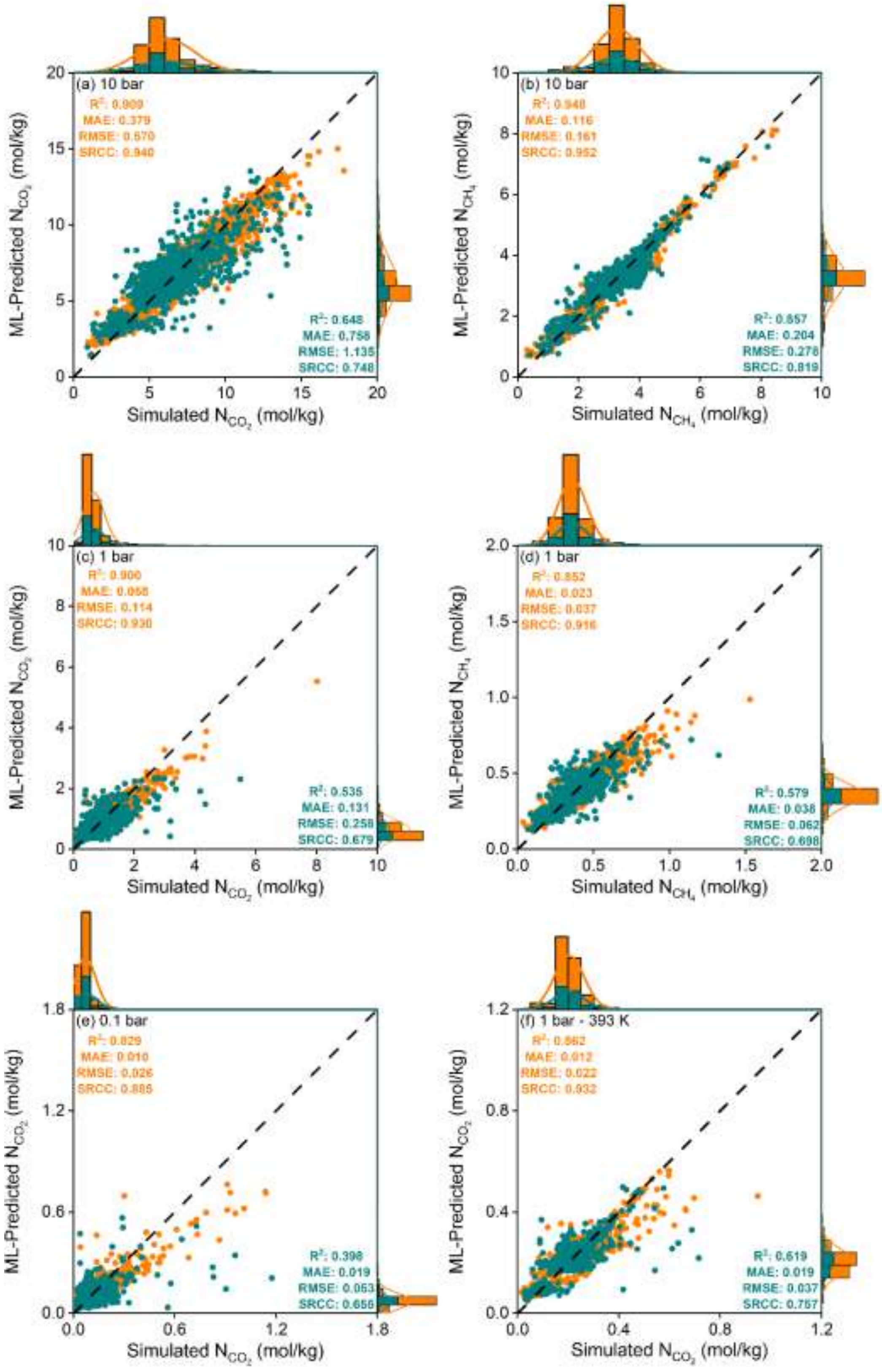

Figure S4. Comparison of CO_2 and CH_4 uptakes predicted by initial ML models constructed with structural descriptors and simulated uptakes in 7340 CoRE COFs and hypoCOFs at 298 K, (a-b) 10 bar, (c-d) 1 bar, (e) 0.1 bar, and (f) only CO_2 uptakes at 1 bar and 393 K.

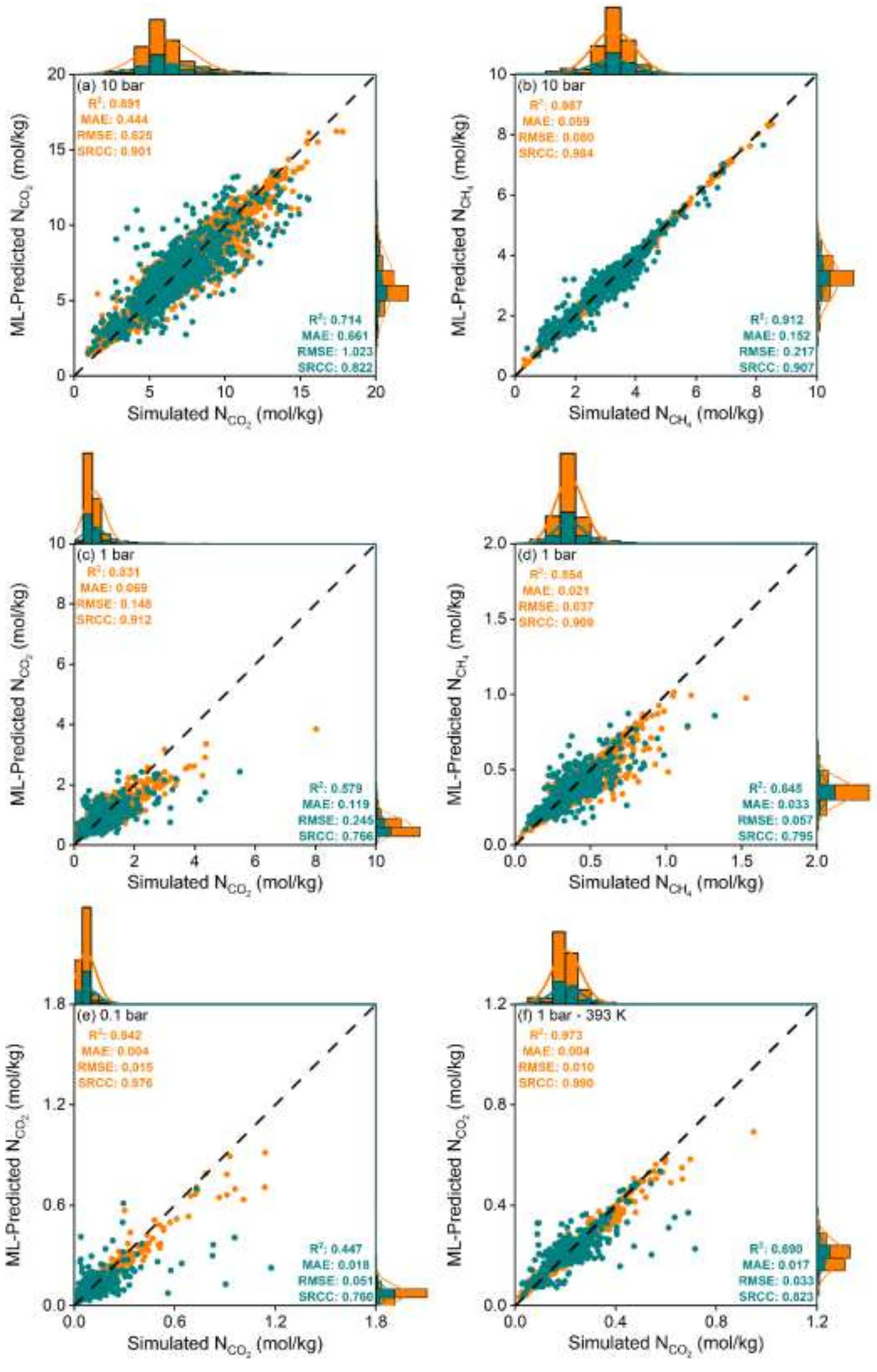

Figure S5. Comparison of CO_2 and CH_4 uptakes predicted by initial ML models constructed with structural, chemical descriptors, and simulated uptakes in 7340 CoRE COFs and hypoCOFs at (a-b) 10 bar, (c-d) 1 bar, (e) 0.1 bar, 298 K, and (f) only CO_2 uptakes at 1 bar and 393 K.

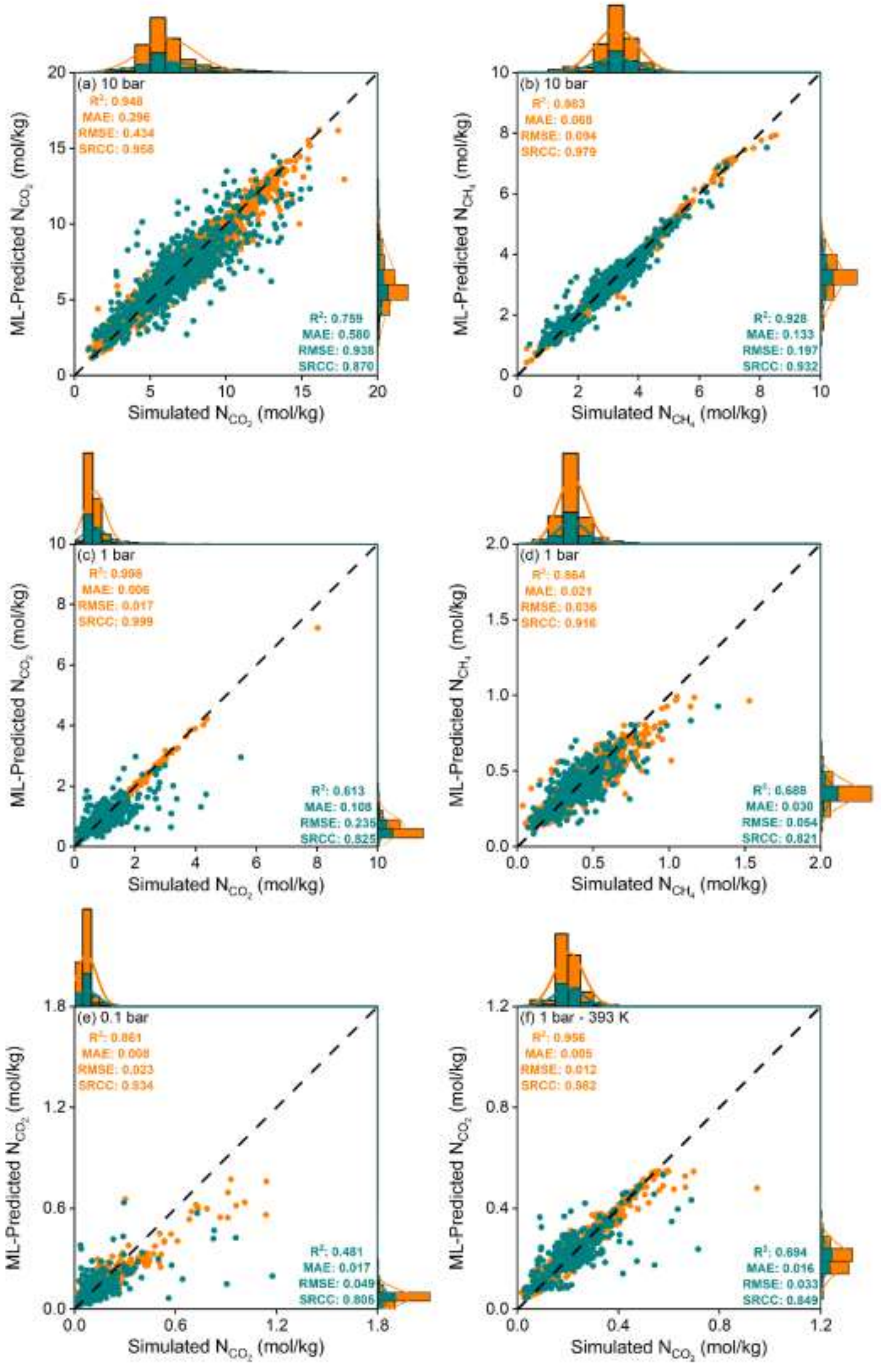

Figure S6. Comparison of CO_2 and CH_4 uptakes predicted by initial ML models constructed with structural, chemical, graph descriptors, and simulated uptakes in 7340 CoRE COFs and hypoCOFs at (a-b) 10 bar, (c-d) 1 bar, (e) 0.1 bar, 298 K, and (f) only CO_2 uptakes at 1 bar and 393 K.

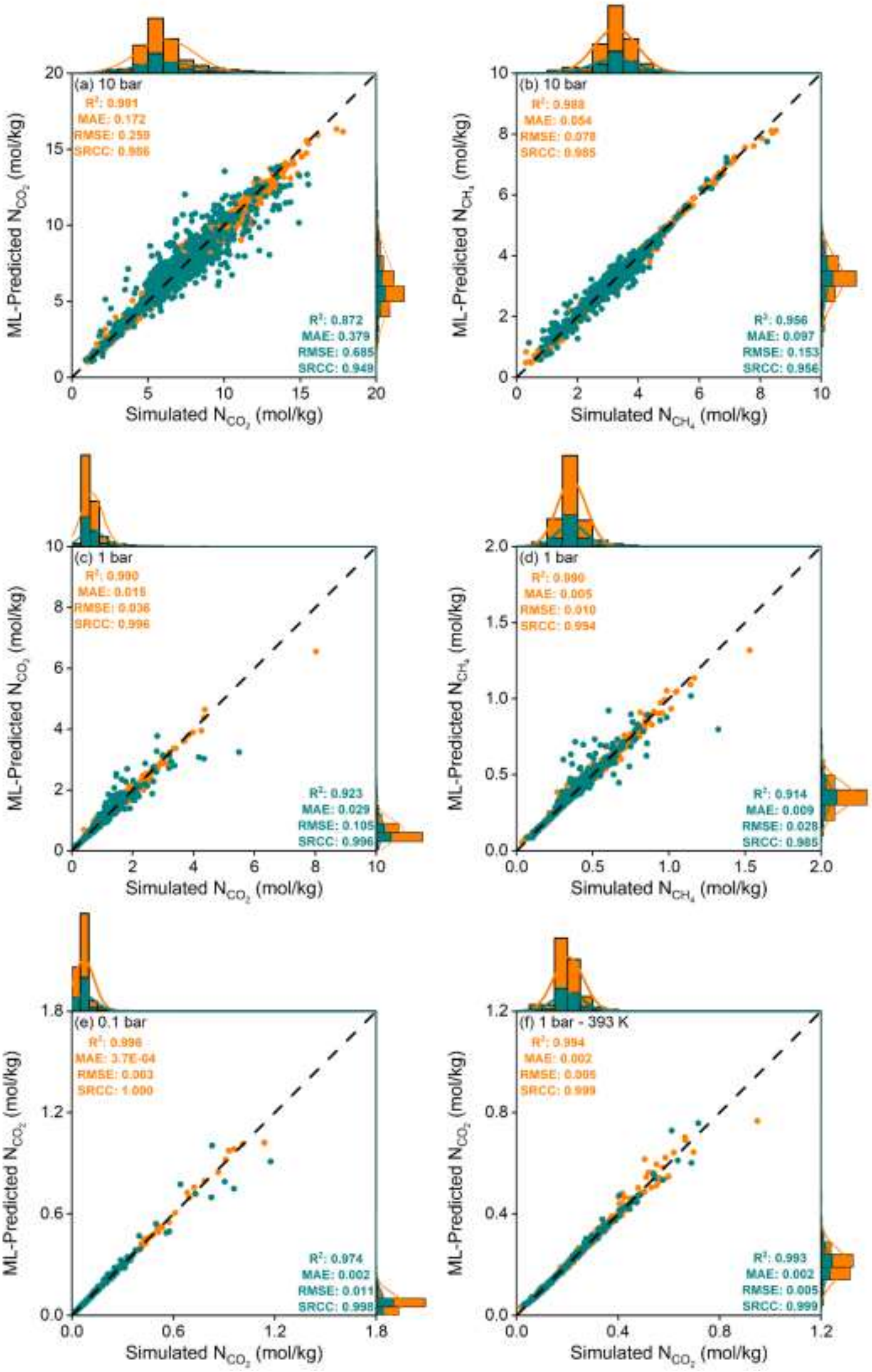

Figure S7. Comparison of CO_2 and CH_4 uptakes predicted by initial ML models constructed with structural, chemical, graph, energy-based descriptors, and simulated uptakes in 7340 CoRE COFs and hypoCOFs at (a-b) 10 bar, (c-d) 1 bar, (e) 0.1 bar, 298 K, and (f) only CO_2 uptakes at 1 bar and 393 K.

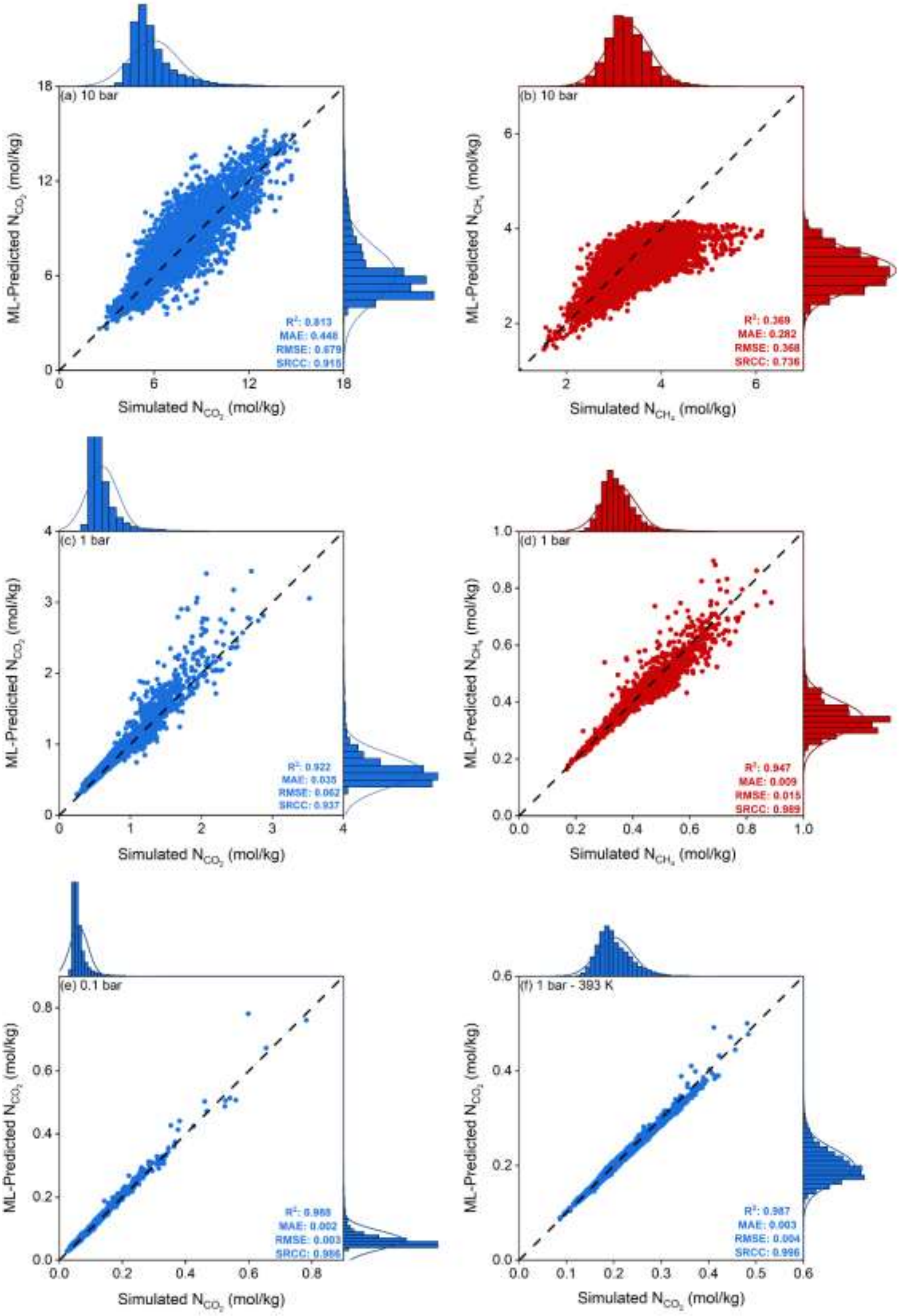

Figure S8. Comparison of CO_2 and CH_4 uptakes predicted by initial ML models constructed with structural, chemical, graph, energy-based descriptors, and simulated uptakes of 19050 hypoCOFs at (a-b) 10 bar, (c-d) 1 bar, (e) 0.1 bar, 298 K, and (f) only CO_2 uptakes at 1 bar and 393 K.

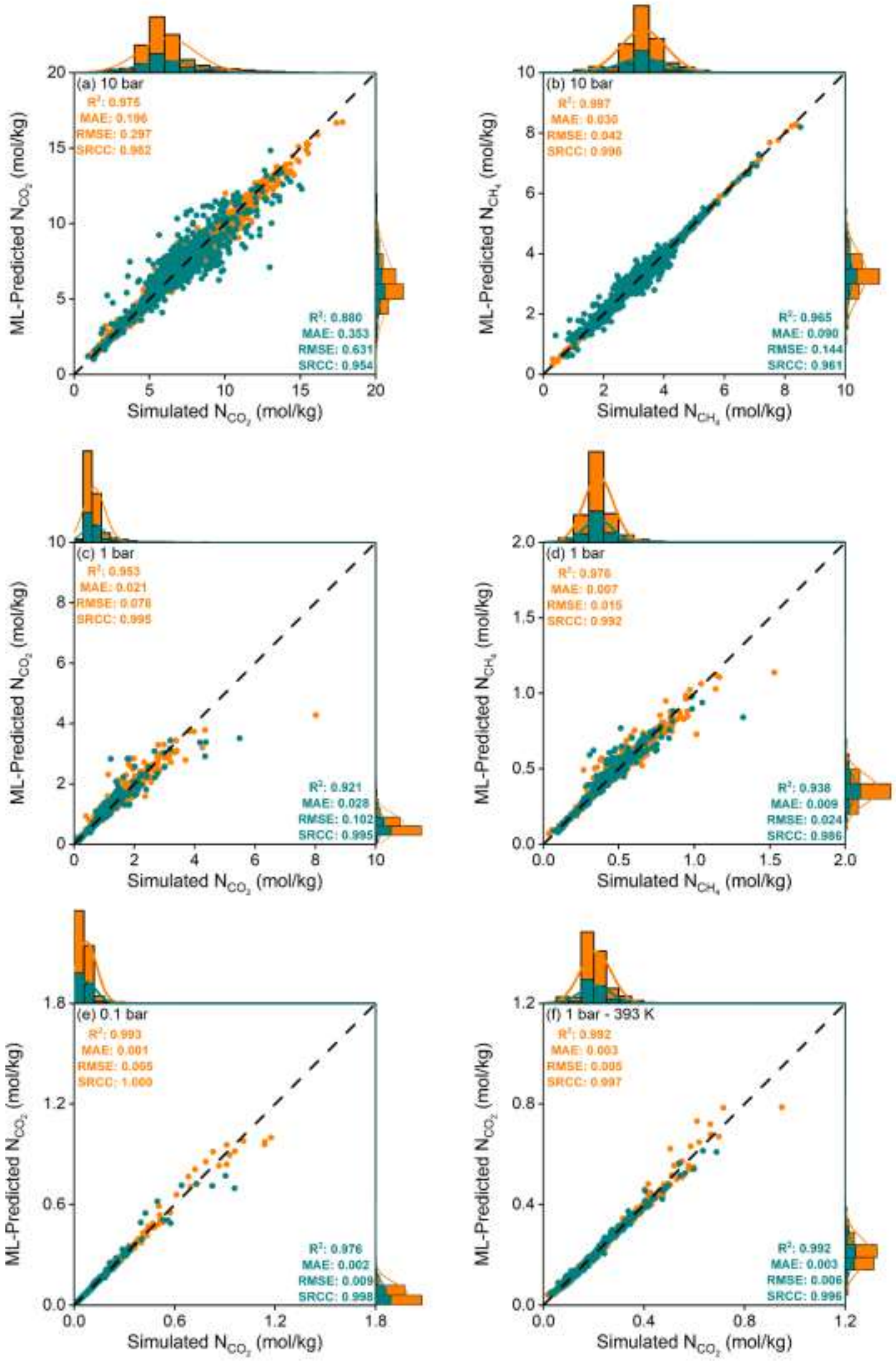

Figure S9. Comparison of CO_2 and CH_4 uptakes predicted by retrained ML models constructed with structural, chemical, graph, energy-based descriptors, and simulated uptakes in 7540 CoRE COFs and hypoCOFs at (a-b) 10 bar, (c-d) 1 bar, (e) 0.1 bar, 298 K, and (f) only CO_2 uptakes at 1 bar and 393 K.

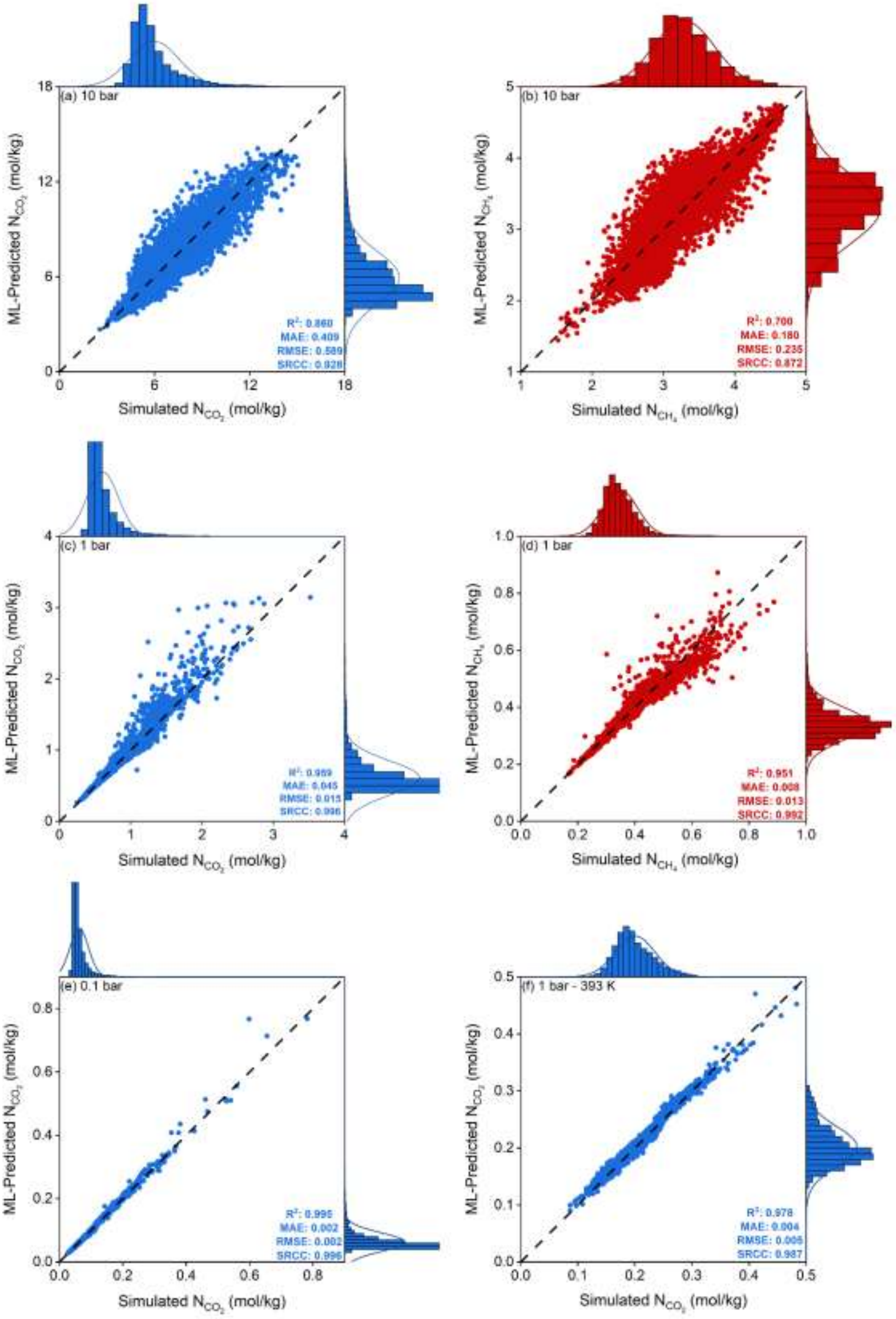

Figure S10. Comparison of CO_2 and CH_4 uptakes predicted by retrained ML models constructed with structural, chemical, graph, energy-based descriptors, and simulated uptakes of 18850 hypoCOFs at (a-b) 10 bar, (c-d) 1 bar, (e) 0.1 bar, 298 K conditions, and (f) only CO_2 uptakes at 1 bar and 393 K.

Table S1. Performance metrics computed to evaluate CoRE COF and hypoCOF adsorbents.

Metric	Formula
Mixture adsorption selectivity	$S_{CO_2/CH_4}=\frac{N_{CO2}}{N_{CH4}}/\frac{y_{CO2}}{y_{CH4}}$
Working capacity (mol/kg)	$\Delta N_{CO2}=N_{ads,CO2}-N_{des,CO2}$
Adsorbent performance score (mol/kg)	$APS=S_{CO_2/CH_4}\times\Delta N_{CO2}$
Percent regenerability	$R\%=\frac{\Delta N_{CO2}}{N_{ads,CO2}}\times 100\%$

$N_{ads,i}$: gas uptake at adsorption condition (mol/kg), $N_{des,i}$: gas uptake at desorption condition (mol/kg), y_i: bulk composition of the gas mixture

Table S2. The list of descriptors to construct machine learning models.

Descriptor Type	Descriptors
Structural (4 descriptors)	Largest Cavity Diameter (Å)<br>Pore Limiting Diameter (Å)<br>Accessible Surface Area (m^2/g)<br>Porosity
Chemical (7 descriptors)	Carbon percentage, C%<br>Hydrogen percentage, H%<br>Nitrogen percentage, N%<br>Oxygen percentage, O%<br>Metalloid Percentage, Me%<br>Ametal Percentage, Ametal%<br>Halogen Percentage, Halogen%
Graph-based (132 descriptors)	Molecular Access Keys (MACCSKeys) Fingerprint Bits
Energy-based (1 descriptor)	Henry's coefficients for CO_2 or CH_4

Table S3. The quantities calculated to evaluate the model accuracies.

Metric	Formula
Coefficient of Determination (R^2)	$1-\frac{1}{M}\sum_{m=1}^{M}(\bar{y}-\hat{y})^2 \ / \frac{1}{M}\sum_{m=1}^{M}(y-\hat{y})^2$
Mean Absolute Error (MAE)	$\sum_{m=1}^{M}\lvert y-\hat{y}\rvert \Big/ M$
Root Mean Square Error (RMSE)	$\sqrt{\sum_{m=1}^{M}(y-\hat{y})^2 \Big/ M}$
Spearman Ranking Correlation Coefficient (SRCC)	$1-\frac{6\sum d_i^2}{M\left(M^2-1\right)}$

M: the number of samples, y: simulated value, $\hat{y}$: predicted value, $\bar{y}$: average of the simulated value, d_i: the difference between the ranks of corresponding variables.

Table S4. The ML pipelines constructed by structural descriptors and their parameters based on the gas adsorption properties of COFs at each adsorption condition.

Property	Best Pipeline with Parameters
10 bar, 298 K (CO_2)	ExtraTreesRegressor(bootstrap=False, max_features=0.7500000000000001, min_samples_leaf=2, min_samples_split=2, n_estimators=100)
1 bar, 298 K (CO_2)	ExtraTreesRegressor(bootstrap=False, max_features=0.55, min_samples_leaf=1, min_samples_split=5, n_estimators=100)
0.1 bar, 298 K (CO_2)	MaxAbsScaler(), RandomForestRegressor(bootstrap=False, max_features=0.15000000000000002, min_samples_leaf=1, min_samples_split=12, n_estimators=100)
10 bar, 298 K (CH_4)	PolynomialFeatures(degree=2, include_bias=False, interaction_only=False), RandomForestRegressor(bootstrap=True, max_features=0.4, min_samples_leaf=2, min_samples_split=9, n_estimators=100)
1 bar, 298 K (CH_4)	Polynomial Features(degree=2, include_bias=False, interaction_only=False), ExtraTreesRegressor(bootstrap=True, max_features=0.7500000000000001, min_samples_leaf=1, min_samples_split=6, n_estimators=100)
1 bar, 393 K (CO_2)	RandomForestRegressor(bootstrap=True, max_features=0.7000000000000001, min_samples_leaf=2, min_samples_split=8, n_estimators=100)

Table S5. The ML pipelines constructed by structural and chemical descriptors and their parameters based on the gas adsorption properties of COFs at each adsorption condition.

Property	Best Pipeline with Parameters
10 bar, 298 K (CO_2)	MaxAbsScaler(), XGBRegressor(learning_rate=0.1, max_depth=6, min_child_weight=4, n_estimators=100, n_jobs=1, objective="reg:squarederror", subsample=0.6500000000000001, verbosity=0)
1 bar, 298 K (CO_2)	StackingEstimator(estimator=RidgeCV()), ExtraTreesRegressor(bootstrap=True, max_features=0.7000000000000001, min_samples_leaf=2, min_samples_split=2, n_estimators=100)
0.1 bar, 298 K (CO_2)	MinMaxScaler(), RandomForestRegressor(bootstrap=False, max_features=0.3, min_samples_leaf=2, min_samples_split=2, n_estimators=100)
10 bar, 298 K (CH_4)	XGBRegressor(learning_rate=0.1, max_depth=9, min_child_weight=1, n_estimators=100, n_jobs=1, objective="reg:squarederror", subsample=0.6000000000000001, verbosity=0)
1 bar, 298 K (CH_4)	GradientBoostingRegressor(alpha=0.9, learning_rate=0.1, loss="huber", max_depth=7, max_features=0.6000000000000001, min_samples_leaf=6, min_samples_split=9, n_estimators=100, subsample=0.9000000000000001)
1 bar, 393 K (CO_2)	StackingEstimator(estimator=RidgeCV()), ExtraTreesRegressor(bootstrap=False, max_features=0.7500000000000001, min_samples_leaf=1, min_samples_split=5, n_estimators=100)

Table S6. The ML pipelines constructed by structural, chemical, and graph-based descriptors and their parameters based on the gas adsorption properties of COFs at each adsorption condition.

Property	Best Pipeline with Parameters
10 bar, 298 K (CO_2)	XGBRegressor(learning_rate=0.1, max_depth=10, min_child_weight=15, n_estimators=100, n_jobs=1, objective="reg:squarederror", subsample=0.7500000000000001, verbosity=0)
1 bar, 298 K (CO_2)	RandomForestRegressor(bootstrap=False, max_features=0.25, min_samples_leaf=1, min_samples_split=3, n_estimators=100)
0.1 bar, 298 K (CO_2)	MaxAbsScaler(), RandomForestRegressor(bootstrap=True, max_features=0.55, min_samples_leaf=1, min_samples_split=7, n_estimators=100)
10 bar, 298 K (CH_4)	XGBRegressor(learning_rate=0.1, max_depth=9, min_child_weight=9, n_estimators=100, n_jobs=1, objective="reg:squarederror", subsample=0.7500000000000001, verbosity=0)
1 bar, 298 K (CH_4)	XGBRegressor(learning_rate=0.1, max_depth=10, min_child_weight=17, n_estimators=100, n_jobs=1, objective="reg:squarederror", subsample=0.5, verbosity=0)
1 bar, 393 K (CO_2)	StackingEstimator(estimator=RandomForestRegressor(bootstrap=True, max_features=0.3, min_samples_leaf=1, min_samples_split=11, n_estimators=100)), FunctionTransformer(copy)), RandomForestRegressor(bootstrap=True, max_features=0.5, min_samples_leaf=6, min_samples_split=11, n_estimators=100)

Table S7. The ML pipelines constructed by structural, chemical, graph- and energy-based descriptors and their parameters based on the gas adsorption properties of COFs at each adsorption condition.

Property	Best Pipeline with Parameters
10 bar, 298 K (CO_2)	ZeroCount(), XGBRegressor(learning_rate=0.1, max_depth=7, min_child_weight=5, n_estimators=100, n_jobs=1, objective="reg:squarederror", subsample=0.6500000000000001, verbosity=0)
1 bar, 298 K (CO_2)	XGBRegressor(learning_rate=0.1, max_depth=5, min_child_weight=2, n_estimators=100, n_jobs=1, objective="reg:squarederror", subsample=0.5, verbosity=0)
0.1 bar, 298 K (CO_2)	ExtraTreesRegressor(bootstrap=False, max_features=0.9000000000000001, min_samples_leaf=1, min_samples_split=6, n_estimators=100)
10 bar, 298 K (CH_4)	XGBRegressor(learning_rate=0.1, max_depth=8, min_child_weight=7, n_estimators=100, n_jobs=1, objective="reg:squarederror", subsample=0.5, verbosity=0)
1 bar, 298 K (CH_4)	XGBRegressor(learning_rate=0.1, max_depth=6, min_child_weight=6, n_estimators=100, n_jobs=1, objective="reg:squarederror", subsample=0.6500000000000001, verbosity=0))
1 bar, 393 K (CO_2)	StackingEstimator(estimator=ExtraTreesRegressor(bootstrap=False, max_features=0.9500000000000001, min_samples_leaf=18, min_samples_split=4, n_estimators=100)), LassoLarsCV(normalize=True)

Table S8. The retrained ML pipelines constructed by structural, chemical, graph- and energy-based descriptors and their parameters based on the gas adsorption properties of COFs at each adsorption condition.

Property	Best Pipeline with Parameters
10 bar, 298 K (CO_2)	XGBRegressor(learning_rate=0.1, max_depth=6, min_child_weight=2, n_estimators=100, n_jobs=1, objective="reg:squarederror", subsample=0.7000000000000001, verbosity=0)
1 bar, 298 K (CO_2)	RobustScaler(),StackingEstimator(estimator=RidgeCV()),XGBRegressor(learning_rate =0.1, max_depth=4, min_child_weight=19, n_estimators=100, n_jobs=1, objective="reg:squarederror", subsample=0.8, verbosity=0))
0.1 bar, 298 K (CO_2)	ExtraTreesRegressor(bootstrap=False, max_features=0.9000000000000001, min_samples_leaf=1, min_samples_split=6, n_estimators=100)
10 bar, 298 K (CH_4)	XGBRegressor(learning_rate=0.1, max_depth=10, min_child_weight=2, n_estimators=100, n_jobs=1, objective="reg:squarederror", subsample=0.5, verbosity=0)
1 bar, 298 K (CH_4)	XGBRegressor(learning_rate=0.1, max_depth=5, min_child_weight=12, n_estimators=100, n_jobs=1, objective="reg:squarederror", subsample=0.6000000000000001, verbosity=0)
1 bar, 393 K (CO_2)	MaxAbsScaler(),StackingEstimator(estimator=RandomForestRegressor(bootstrap=True, max_features=0.15000000000000002, min_samples_leaf=12, in_samples_split=13, n_estimators=100)), RidgeCV()

Table S9. Separation performances of the top 10 hypoCOFs for the most optimal PTSA process.

ID	APS (mol/kg)	S_{CO_2/CH_4}	R%	LCD (Å)	PLD (Å)	ϕ	S_{acc} (m^2/g)
linker110_C_linker86_C_sod_relaxed	164.18	11.49	95.90	11.94	6.05	0.66	2880.1
linker92_C_linker91_C_bpb_relaxed	105.18	7.26	93.86	7.24	7.14	0.82	3348.2
linker110_C_linker54_C_lcs_relaxed	88.99	8.39	96.46	8.82	3.81	0.56	1793.5
linker91_C_linker91_C_acs-g_relaxed	83.87	6.88	95.60	7.41	6.43	0.79	2834.1
linker92_C_linker91_C_etg_relaxed	81.65	5.21	97.08	9.22	7.82	0.84	3496.7
linker105_C_linker92_C_sur_relaxed	74.70	5.64	96.75	7.67	5.95	0.73	3388.6
linker105_C_linker93_C_hst_relaxed_interp_2	74.60	5.35	97.18	6.96	4.81	0.69	3640.9
linker104_NH_linker86_CO_uoo_relaxed	74.00	5.61	98.31	11.77	8.06	0.78	4230.3
linker99_C_linker76_C_qtz_relaxed	73.42	4.30	98.25	7.62	6.41	0.82	5190.4
linker108_C_linker40_C_tcb_relaxed	72.59	4.82	96.94	9.31	7.96	0.83	4581.0

Table S10. Separation performances of the top 10 CoRE COFs for the most optimal PTSA process.

ID	APS (mol/kg)	S_{CO_2/CH_4}	R%	LCD (Å)	PLD (Å)	ϕ	S_{acc} (m^2/g)
92	77.61	4.42	98.65	9.04	7.99	0.78	5128.7
368	71.79	7.87	97.79	17.69	17.43	0.70	1446.4
70	70.79	4.95	95.75	9.35	7.17	0.75	4355.2
455	70.46	4.98	95.30	24.86	24.30	0.87	4905.4
370	69.25	6.97	97.72	20.86	20.63	0.74	2091.0
468	67.47	7.25	95.17	5.58	4.44	0.63	1898.1
52	60.08	5.01	97.43	10.16	9.73	0.72	3541.2
290	59.47	5.47	96.80	13.44	13.27	0.74	3224.9
374	54.92	4.12	97.93	11.20	8.17	0.76	4119.9
516	53.58	4.78	97.19	11.53	11.16	0.77	4582.3

Table S11. 8 most common bits of COF "skeleton" together with their SMARTS Keys, explanations and depictions from MACCSKeys molecular fingerprint library, respectively.

Bit Name	SMARTS Keys	Explanation	Depiction
105	('*@*(@*)@*',0)	A sequence of atoms connected by aromatic bonds, with one atom being part of an aromatic ring.	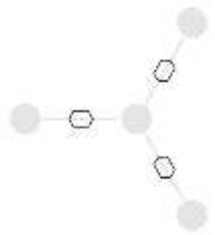
131	([!#6;!#1;!H0]',1)	More than one atom that is not carbon and not hydrogen, and must have at least one hydrogen atom attached.	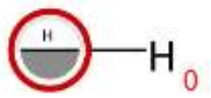
141	('[CH3]',2)	More than two methyl groups in the structure.	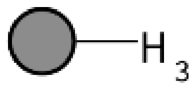
145	('*1~*~*~*~*~*~1',1)	More than one six-membered rings in the structure.	
149	('[C;H3,H4]',1)	More than one carbon atom that is bonded to either three or four hydrogen atoms.	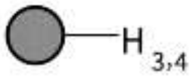
160	('[C;H3,H4]',0)	Carbon atom that is bonded to either three or four hydrogen atoms.	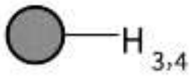
163	('*1~*~*~*~*~*~1',0)	A six-membered ring structure in a molecule, where each atom in the ring can be of any type and the bonds.	
165	('[R]',0)	Any ring atom in a molecular structure.	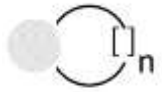
